# MHConstructor: a high-throughput, haplotype-informed solution to the MHC assembly challenge

**DOI:** 10.1186/s13059-024-03412-6

**Published:** 2024-10-17

**Authors:** Kristen J. Wade, Rayo Suseno, Kerry Kizer, Jacqueline Williams, Juliano Boquett, Stacy Caillier, Nicholas R. Pollock, Adam Renschen, Adam Santaniello, Jorge R. Oksenberg, Paul J. Norman, Danillo G. Augusto, Jill A. Hollenbach

**Affiliations:** 1 Department of Neurology, Weill Institute for Neurosciences, University of California San Francisco, San Francisco, CA USA; 2 Department of Biomedical Informatics, Anschutz Medical Campus, University of Colorado, Aurora, CO USA; 3 Department of Immunology and Microbiology, Anschutz Medical Campus, University of Colorado, Aurora, CO USA; 4 Department of Biological Sciences, University of North Carolina Charlotte, Charlotte, NC USA; 5 Programa de Pós-Graduação em Genética, Universidade Federal do Paraná, Curitiba, Brazil; 6 Department of Epidemiology and Biostatistics, University of California San Francisco, San Francisco, CA USA

**Keywords:** Major histocompatibility complex, Human leukocyte antigen genes, Haplotype, De novo assembly, Short-read sequencing

## Abstract

The extremely high levels of genetic polymorphism within the human major histocompatibility complex (MHC) limit the usefulness of reference-based alignment methods for sequence assembly. We incorporate a short-read, de novo assembly algorithm into a workflow for novel application to the MHC. *MHConstructor* is a containerized pipeline designed for high-throughput, haplotype-informed, reproducible assembly of both whole genome sequencing and target capture short-read data in large, population cohorts. To-date, no other self-contained tool exists for the generation of de novo MHC assemblies from short-read data. *MHConstructor* facilitates wide-spread access to high-quality, alignment-free MHC sequence analysis.

**Supplementary Information:**

The online version contains supplementary material available at 10.1186/s13059-024-03412-6.

## Background

The vast majority of human diseases have a complex, polygenic component, with risk loci often spread throughout the genome [1]. Notably, the single region of the human genome with the greatest number of trait and disease association signals is the major histocompatibility complex (MHC) [2]. The MHC is located on the short arm of chromosome 6, at 6p21.31. MHC genomic variation is strongly associated with its role in the adaptive immune system primarily due to the presence of the human leukocyte antigen (HLA) genes [3, 4]. HLA class I and class II genes encode the antigen-presenting, surface-marker proteins responsible for driving the adaptive immune responses. While most disease association studies have focused on variation in HLA, the extended MHC region contains over 165 protein-coding gene, many involved in the immune response [5]. Over evolutionary time, immune function across the MHC has been subjected to a diversity of selective scenarios, ultimately producing the single most polymorphic region of the human genome [3, 6–14].

Extreme polymorphism has made it difficult to thoroughly elucidate the molecular mechanisms by which MHC variation contributes to numerous diseases. Advances toward this end have been primarily driven by targeted analysis of protein-coding genes or genome-wide association studies (GWAS) conducted with single-nucleotide polymorphism (SNP) arrays [2, 15–18]. While many disease-associated SNPs map to the classical HLA genes, non-HLA associations throughout the MHC have also been identified (International MHC and Autoimmunity Genetics Network (IMAGEN) et al. [19–22]. However, our current understanding of the role of MHC variation in disease remains limited since this region is often excluded from whole genome analysis, which provides greater resolution variant maps than SNP arrays. This is not for lack of interest in examining high-resolution nucleotide variation, but due to the significant challenges inherent to a robust assembly of short-read data across the 5 Mbp MHC region. In particular, novel heterozygosity, copy number variation, and large structural variants are challenging to identify using methods that merely align sequencing reads to a reference sequence [23].

Although long-read technology is becoming the industry standard for high-quality reference genomes [24, 25], its applicability to population-level research, such as large (*n* ≥ 500) disease association and demographic studies, remains limited due to computational complexity and cost of long read sequencing at population scale. To handle the diverse collection of challenges involved in high-throughput, short-read assembly and variant calling for the MHC, we have developed a de novo assembly pipeline, designed specifically for the extended, 5-mb MHC region. We have built the pipeline into a *Singularity* container [26], so that users can simply download the container, build the image, and reproducibly run the pipeline on large, trait-association cohorts. We have named this tool *MHConstructor* [27].

*MHConstructor* provides solutions to four main challenges of MHC assembly with short-read data:

1. Genotyping of the HLA genes is notoriously difficult with short-read data, due to the high sequence similarity between HLA paralogs and difficulty assigning reads [28]. Since many dedicated tools have already been published to handle this challenge, we have included one of these, *T1K* [29], into the *MHConstructor* pipeline to generate HLA genotypes at first field resolution.
2. Large structural variation associated with the MHC is difficult to capture with short-read assembly [23, 30]. There are two primary loci contributing to the majority of observed, large structural variation at the MHC: the HLA class II region and copy number variation associated with the C4 gene [24, 31]. To handle diploid, structural variation at the HLA class II region, *MHConstructor* performs haplotype binning by separately assembling reads attributable to heterozygous HLA class II haplotypes. Haplotype binning has already been successfully used in several MHC assembly contexts [28, 32]. Furthermore, to maintain the correctly assembled HLA class II structure for downstream analysis, *MHConstructor* generates intermediate consensus sequences, by scaffolding assemblies to their relevant haplotype reference [24]. Copy number and genotyping of C4A/B are accounted for by including our tool *C4Investigator* [33] into the *MHConstructor* pipeline.
3. The MHC is the most polymorphic region of the human genome [34, 35]. Therefore, methods which describe variation merely by aligning sequence reads to a reference genome are likely to miss much of this novel variation that does not occur in the reference genome [23]. De novo sequence assembly is a commonly used method of reference-free sequence assembly and has already been successfully used to generate high-quality short-read MHC assemblies [31, 32, 36–38]. It functions by breaking sequencing reads into smaller sequence fragments of length *k* (kmers) and then connecting kmers into graph structures based on sequence overlap [39, 40]. These structures are known as De Bruijn graphs and represent the most likely path through overlapping sequence fragments [39, 40]. These graphs are then condensed into longer assembled sequence fragments known as contigs. The primary benefit of this approach is that it does not rely on alignment to a reference sequence and therefore is much more likely to capture previously undescribed variation [23, 40–43]
4. While many previous advances have been made in handling the problem of MHC sequence assembly, to-date, none have been developed into a self-contained tool that can be used reproducibly or that can scale to large, population-level cohorts. The large number of software and associated dependencies required to perform de novo assembly alone often creates a barrier to accessibility and reproducibility, particularly as version control is widely inconsistent across bioinformatic tools [44–46]. These challenges have resulted in limited capacity to reliably generate MHC sequences and thoroughly query the functional role of MHC variation in disease association contexts.

Even with all that is currently known of MHC diversity, it remains an underestimation of the true extent of polymorphism within this complex genome region. *MHConstructor* reduces the complexity of the de novo assembly process while increasing reproducibility and, in doing so, provides access to a fuller spectrum of MHC diversity. With *MHConstructor*, we have also contributed MHC assemblies for an additional 369 new individuals. This work has the potential to improve our understanding of genetic variation in this functionally significant, yet understudied region of the human genome and its role in human health and disease.

## Results

### Haplotype-guided, de novo short-read MHC assembly workflow

**Fig. 1:**
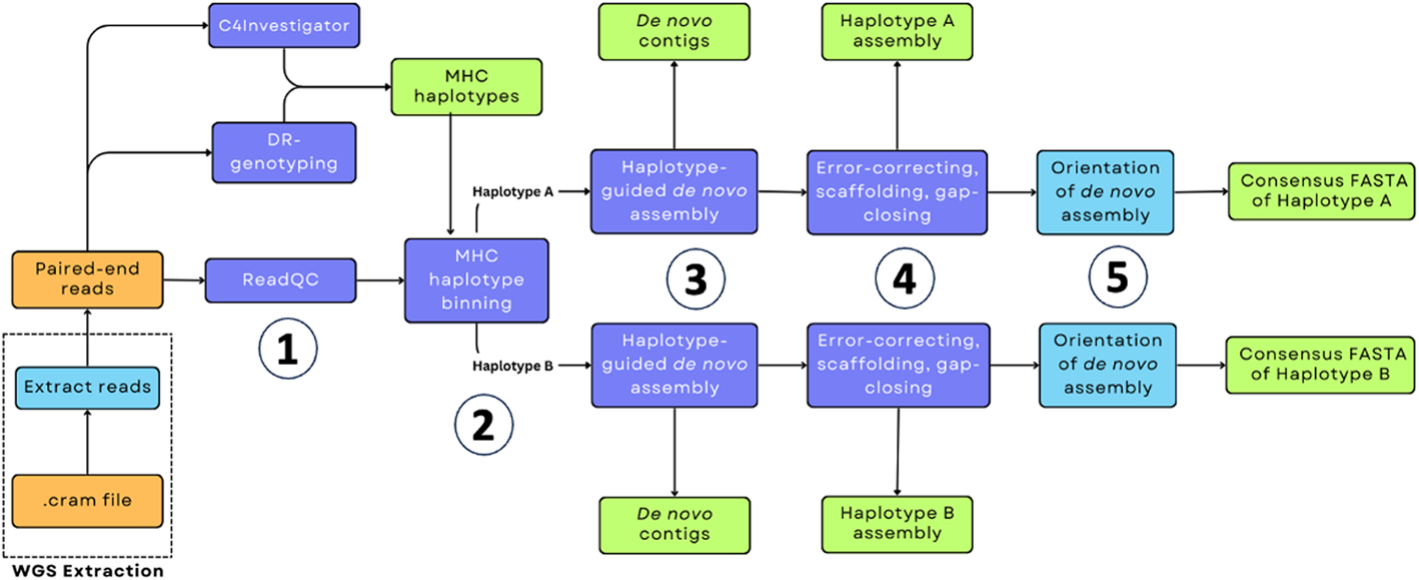
*MHConstructor* workflow for haplotype-informed, de novo MHC assembly. Orange indicates source data. Purple indicates previously published software. Teal indicates novel software. Green indicates output data

*MHConstructor* is a short-read de novo assembly tool designed specifically for application to the MHC region of the genome. We implement and adapt a previously established pipeline, which was originally designed in a comparative species context to capture novel, species-specific sequence when a reference genome was not available for alignment [43]. Instead of relying on a single, guide sequence from one reference genome, we choose guide sequences that represent the six, high-quality major MHC reference sequences [24]. These MHC references represent the major categories of known HLA class II structural variation. We describe these as “Best-matched MHC Haplotypes (or BMHs)” (Additional files 1 and 2) and assign each individual either one (homozygous BMH) or two (heterozygous BMHs), based on their *HLA*-DRB1 genotype. Each major *HLA*-DRB1 allele is associated with one of the six structural haplotypes [24, 38]. These guide MHC haplotypes are used to facilitate haplotype-guided, de novo assembly, according to the algorithm of Lischer and Shimizu [43]. This strategy provides a convenient framework for handling heterozygous, diploid sequencing data in a haplotype-informed manner that can handle the large, structural variation of the HLA class II region. We then carry out the de novo assembly process in a haplotype-informed manner, generating pseudo-haploid assemblies, rather than a combined diploid assembly, for each individual (Fig. 1).

### Evaluation of assembly performance metrics

#### Assembly speed and quality are impacted by assembly kmer size and sequencing depth

De novo assembly algorithms are inherently computationally intensive, which can introduce implementation challenges in a high-throughput context. To characterize how our algorithm responds to various parametric spaces, we have tested assembly parameters on a high-performance computing cluster and describe the relationship of key assembly parameters, assembly kmer size, and sequencing depth to overall performance. Small kmer sizes are considered more successful at high accuracy mapping, whereas longer kmer sizes can improve repeat content assembly. To generate an initial range of starting kmer sizes to evaluate, *KmerGen*ie was applied to a set of *n* = 20 samples, assessing the mapped reads vs unmapped reads separately. Higher kmer values were found to be more relevant for reads which were able to be mapped to the haplotype guide sequence (*k* = 41–61) (Table S1), while lower sizes of *k* were predicted for the unmapped reads (*k* = 21–26) (Table S2). Kmer size predictions for unmappable reads showed less variation between individuals than the mappable reads. For reads which mapped to the guide haplotype, best kmer size varied significantly across samples (stdev 4.15–21.19). Additionally, the number of reads evaluated impacts the best kmer size identified, with greater sequencing depth allowing for larger kmer sizes. However, variability in predicted best kmer size between samples also increases with greater sequencing depth. We hypothesize that this may be driven by the increased probability of including reads representing variants that may be unique to each individual associated with greater coverage depth. Therefore, the more reads, the more variability in best predicted kmer size, as each individual may have unique patterns of variation that lower coverage sampling may not identify. Overall, there is less variability in kmer prediction for reads which do not initially map to guide haplotype, and these values are generally lower than those predicted for the well mapped reads, consistently falling between *k* = 21 and *k* = 26 for all sequencing depths considered (Table S2).

We empirically tested de novo assembly kmer size by evaluating assembly quality metrics for varying kmer sizes and find that using larger kmer sizes for mapped reads produce stronger assembly metrics (Fig. S1). Additionally, we measured how assembly speed is impacted by choice of kmer size. When the kmer size used to assemble unmapped reads is small (< 30), user time for the entire analysis increases in an approximately exponential scale with respect to starting read count (Table S3). However, at *k* = 51, for both unmapped reads and mapped reads, the relationship between starting read count and analysis time becomes linear (Table S4). For this reason, though *KmerGen*ie predicted a smaller kmer size to be most accurate for the unmappable reads, we chose to use larger values in the interest of speed. Using the above metrics, we chose *k* = 51 for de novo assembly of both haplotype-mapping and non-mapping reads. Unsurprisingly, we find that better assembly metrics are correlated with higher sequencing depth (Fig. S2). However, we do find that total length of de novo assembled contig sequence generated reaches an upper limit at around ~ 4.6 Mb. This may be indicative of the upper limit of sequence that can be de novo assembled with short-read data. We find that for target capture samples with an average sequencing depth of 60X, the full analysis takes approximately one and a half hours to complete per MHC haplotype, with multithreading capacity (Table 1). The WGS 30X 1000GenomesProject samples, however, were much quicker to assemble, with an average time of 26 min per MHC haplotype (Table 1).

**Table 1:** Average runtime (in user minutes) for *MHConstructor* assembly, at eight threads on high-performance computing cluster

Sequencing depth	de novo assembly (min)	Error correction + scaffolding (min)	Total assembly run (min)
65X target capture	53	51	104
30X WGS	13	13	26

### Quantification of MHConstructor error rates using high-quality MHC reference sequences

#### Haplotype-informed assembly validation via re-creation of known, fully characterized MHC sequences

In addition to quantifying overall assembly quality metrics, we also described the error rate inherent to the short-read assembly process. We first evaluated this using the set of high-quality references representing haploid MHC sequences which have also been incorporated into *MHConstructor* as guide sequences [24]. Using the Illumina short-read dataset associated with each of the haploid MHC reference sequences (*SRA:* SRP348947, *BioProject:* PRJNA764575) [24], we re-assembled each sequence de novo using *MHConstructor*. Summary statistics for these assemblies can be found in Table S5. Reference MHC coverage was incomplete (Tables S6, S7). However, based on our findings regarding the impact of sequencing coverage depth on assembly quality described in the “Assembly speed and quality are impacted by assembly kmer size and sequencing depth” section, we inferred that this was due to the low starting read count (Table S5), corresponding to a < 30X average coverage across the MHC, limiting our ability to recover the full region. Therefore, we calculated the error statistics as a percent of the total assembled sequence, rather than as a percentage of the entire length of the MHC. We found that the average percent error for base calling (base call error %) was 0.031%, the average error attributable to non-repeat associated false SVs was 0.041%, and the average error attributable to repeat-derived false SVs was 0.05% of the total assembled sequence for each haplotype (Table 2).

**Table 2:** Target capture MHC assemblies exhibit 0.119% average total error in homozygous cell lines, SV ≥ 1 bp

MHC haplotype	Total assembly length (bp)	Base call error (%)	Non-repeat associated SV error (%)	Repeat-associated SV error (%)	Total error (%)
OK649231	4,127,329	0.0267	0.0350	0.0899	0.1516
OK649232	3,908,195	0.0311	0.0515	0.1144	0.1970
OK649234	4,539,318	0.0218	0.0441	0.0068	0.0727
OK649235	3,710,459	0.0554	0.0215	0.0124	0.0893
OK649236	4,196,340	0.0211	0.0528	0.0080	0.0819

SV: structural variant >= 1bp in length

#### Use of phased, long-read MHC haplotype sequences to determine diploid de novo assembly error rate

To further validate this method and ensure that it can successfully handle the heterozygous, diploid sequence data found in human populations, we used *MHConstructor* to re-assemble phased, diploid MHC reference sequences [47] from corresponding 1000 Genomes Project 30X WGS reads. We generated the assemblies using the long-read sequenced, phased haplotype sequence as the guide sequence. We find that no large structural variants (> 1 kb) are erroneously introduced (Fig. S3) when using a threshold on the Ragtag [48] scaffold orientation, which filters out any scaffolds that have a location score < 0.1 or location score < 0.3 and an orientation score < 0.75. In order to evaluate nucleotide composition error, we aligned *MHConstructor* consensus haplotypes to their long-read, phased haplotypes using *minimap2* to describe base call errors [49] and *Assemblytics* to identify structural errors (≥ 1 bp INDELs) [50]. The amount of mismatched sequence between the two was determined by counting the number of variants identified and dividing by the total length of the *MHConstructor* consensus haplotype. We found that the average percent error for base calling (base call error %) was 0.17%, the average error attributable to non-repeat associated false structural variants (SVs) (> 1 bp) was 0.18%, and the average error attributable to repeat-derived false SVs was 0.1% of the total assembled sequence for each haplotype (Table 3). Assessment of the categories and sizes of false structural variants (> 1 bp) revealed low frequency of repeat expansions and repeat contractions. Across the six MHC haplotypes evaluated, we only found three instances of false structural variants greater than or equal to 500 bp in length. Two instances were caused by a 630-bp and 646-bp repeat expansions, and one was caused by a 2842-bp repeat contraction (Fig. S4-S6). Otherwise, all false SVs identified were under 500 bp in length (Fig. S4-S6). We found that of the sites in the *MHConstructor* assembled haplotypes that did not match the phased, long-read haplotype guide sequence, between 34 and 54% were a result of true assembly error, with the remaining sites caused by improper inclusion of a heterozygous allele attributed to the other phased haplotype (Table S8).

**Table 3:** MHC assemblies from WGS data exhibit an average of 0.44% total error in 1000GenomesProject individuals, SV ≥ 1 bp

MHC haplotype	Total assembly length (bp)	Base call error (%)	Non-repeat associated SV error (%)	Repeat-associated SV error (%)	Total error (%)
HG00621.1	4,684,211	0.136	0.188	0.089	0.414
HG00621.2	4,654,849	0.162	0.162	0.107	0.430
NA19240.1	4,739,094	0.179	0.162	0.065	0.407
NA19240.2	4,728,464	0.211	0.160	0.072	0.442
NA20129.1	4,713,830	0.145	0.186	0.177	0.509
NA20129.2	4,538,560	0.162	0.195	0.058	0.415

SV: structural variant >= 1bp in length

#### MHConstructor is sensitive to novel nucleotide variation not observed in the guide sequences

Though de novo assembly methods are known to be a reliable, reference-free method of capturing novel sequence variation [39, 40], we validated this characteristic using ART [51] to simulate Illumina NGS sequencing reads for assembly with *MHConstructor*. Simulated reads were derived from the fully phased MHC haplotype sequences of HLA class II heterozygous individuals HG00621 and NA19240, described in the “Use of phased, long-read MHC haplotype sequences to determine diploid de novo assembly error rate” section [47]. To evaluate *MHConstructor* performance in the simpler context of recreating a single MHC haplotype sequence, we generated a set of synthetic 30X coverage short reads from each ground truth haplotype sequence individually, representing haploid sequence data. To represent realistic, diploid sequencing data, we generated a set of reads from both haplotype sequences combined, to represent diploid sequence at 30X coverage. The set of novel single-nucleotide variants (SNVs) and structural variants (SVs) for each ground truth MHC haplotype sequence was established by aligning each to their corresponding BMH guide sequence. Positions annotated with variation from the guide sequence were designated novel variation. Following *MHConstructor* assembly, we find that for individual HG00621, 99.3–99.5% and 99.1–99.5% of novel SNVs were correctly assembled from the haploid and diploid simulated read sets, respectively (Table S9). For individual NA19240, 97.5–98.4% and 98–98.2% of novel SNVs were correctly assembled from the haploid and diploid simulated read sets, respectively (Table S9). *MHConstructor* also exhibited high accuracy of correctly identifying novel SVs. For individual HG00621, 99.6% and 86.2–98.8% of novel SVs were correctly assembled from the haploid and diploid simulated read sets, respectively (Table S10). For individual NA19240, 99.4–99.5% and 93.7–99.0% of novel SVs were correctly assembled from the haploid and diploid simulated read sets, respectively (Table S10). In both individuals, SNV assembly exhibited consistently high recovery of novel variation (> 97.5%). SV assembly quality was also high for haploid assemblies but showed a slightly wider range for the diploid assemblies (86.2–99.0%).

#### HLA class II haplotype-defining structural variation is correctly assembled

**Fig. 2:**
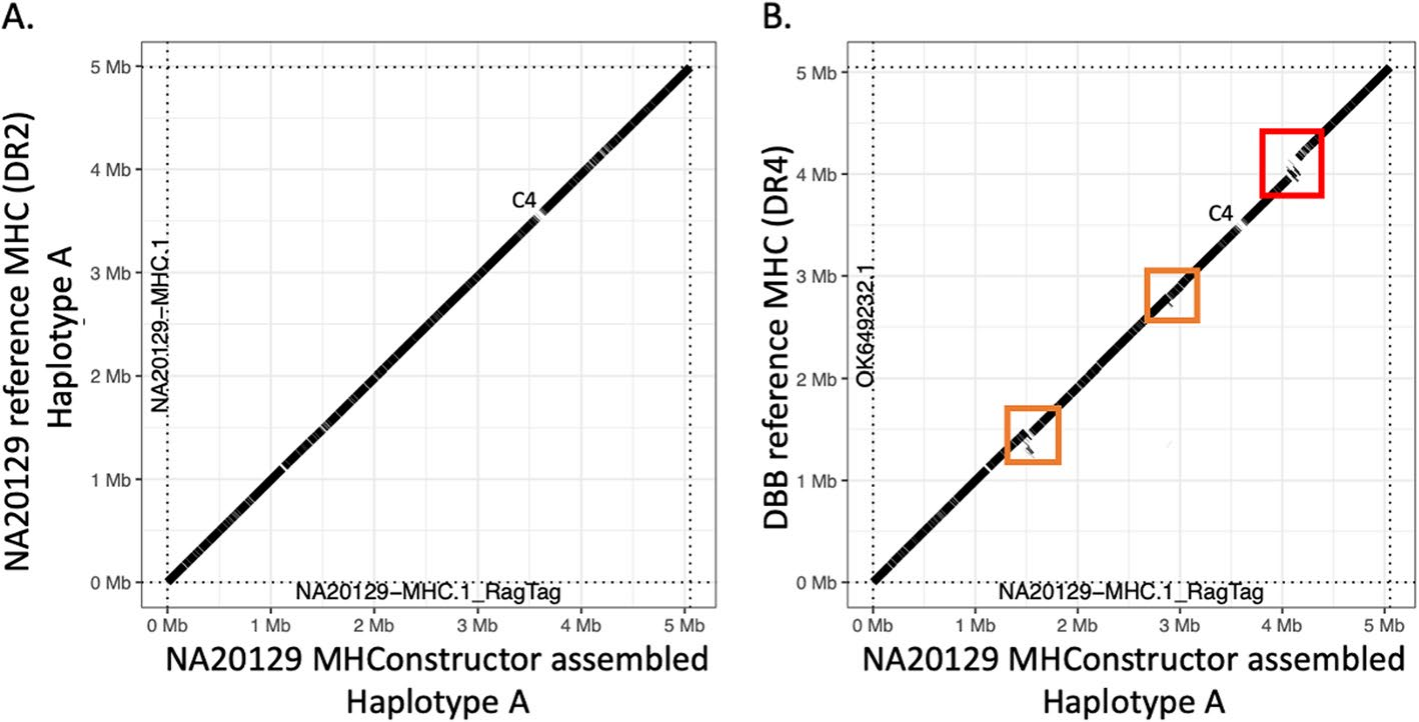
HLA class II structural variation is correctly assembled. Dot plots depict the *MHConstructor*, haplotype consensus sequence for 1000 Genomes Project ASW individual NA20129, aligned to **A** the correct, phased reference haplotype, which has the short, DR3 HLA class II status and **B** to reference haplotype DBB, which has the long, DR4 HLA class II status. Solid diagonal line indicates continuous alignment. Breaks in the line indicate assembly gaps. The red box indicates structural variation resulting from mis-matched HLA class II status. Orange boxes indicate additional regions exhibiting structural variation. The position corresponding to C4 is designated

The six primary MHC haplotypes used here as guide sequences are distinguished primarily by structural variation in the HLA class II region. This variation is primarily driven by *HLA-DRB1*; its three duplication paralogs, *HLA-DRB3*, *HLA-DRB4*, and *HLA-DRB5*; and several pseudogenes. Most individuals have only partial representation of these. The generally observed combinations are *HLA-DRB1* with *HLA-DRB5* (DR2), *HLA-DRB1* with *HLA-DRB3* (DR3), and *HLA-DRB1* with *HLA- DRB4* (DR4) [52, 53]. Of these three, the DR4 haplotype contains extra sequence content between *HLA-DRB4* and *HLA-DRB1*, thus extending the length of the MHC and providing a challenge to correct sequence assembly. To ensure that our de novo assembly method is capable of properly capturing this structural variation, we assessed the reconstruction of structural variation associated with the HLA-DRB1, HLA-DRB3, HLA-DRB4, and HLA-DRB5 genes via dot plot visualization of oriented de novo assemblies aligned to the MHC phased reference sequences (Fig. 2), using the R package *paftools*. When assemblies are aligned to the guide BMH sequence with their correctly matched DR-status, there is continuous alignment over the class II region (Fig. 2A). Conversely, when aligned to a guide BMH with a mismatched DR-status (Fig. 2B), a large break in the alignment is observed, corresponding to the length of the expected structural variation. We find that our approach is capable of correctly assembling this structurally varied region. This was additionally confirmed through visual inspection in Mauve (Fig. S7), which we demonstrate with an example from an individual with European ancestry (individual Z) who is heterozygous for DR3 and DR4. Two Individual Z consensus sequences were generated by aligning the de novo assembly to the APD (DR3) and DBB (DR4) reference sequences separately. Next, these alignments were visualized in Mauve [54]. The absence of sequence gaps in the alignment shows that the structure was correctly assembled in both cases. Promisingly, we find that even in the event of choosing a mismatched guide MHC for the assembly process (i.e., DR4 instead of DR2), the correct class II structure will still be constructed, as long as assembly is scaffolded with respect to the correct, DR-matched guide, though with lower resolution with respect to identification of novel variation (Fig. S8).

#### Assembly of short-read data reliably describes repetitive element family composition

Since repetitive elements are known to comprise 50–52% of the MHC [24] and roughly 60% of the genome as a whole [55], we wanted to ensure that this method was reliably assembling genomic sequence composed of repetitive content. All final assembled sequences were annotated with the RepeatMasker (RM) software, using default settings (www.repeatmasker.org). We then compared our novel assembly RM predictions to RM predictions for the original, phased, MHC reference sequences. We find that repeat element annotations are highly syntenic and differ only by ~ 3% of total annotated repeat content (Fig. S9). This demonstrates that our assembly method can properly handle short-read data and sensitively assemble the sequences of repetitive elements in the MHC with good accuracy at the level of repetitive element families.

### *MHConstructor* assembly of target capture 60 × and 1000 Genomes Project 30 × WGS short-read MHC data

#### Haplotype-guided, de novo assembly generates high-quality extended MHC assemblies

*MHConstructor* was then used to generate 536 novel MHC haplotype assemblies from MHC target capture short-read sequencing data. Assembly quality is generally evaluated according to several primary metrics. N50 is a traditional indicator of assembly quality, as it provides a description of contig size distribution, with a larger N50 indicating more contigs of greater length. However, this metric is now recognized by the bioinformatics community to not be wholly representative of assembly quality, especially in genomic regions with complex sequence content. Therefore, we also describe the number of contigs per assembly as well assembly length (Additional files 3 and 4). In order to use these scores to meaningfully evaluate the performance of the reference-guided, de novo assembly method, we compared them to a previously published dataset of short-read, de novo assembled MHC sequences, *n* = 95 haplotypes [38], based on assembled contigs, prior to scaffolding. For the target capture assemblies (*n* = 536 haplotypes), we find that while the distributions show much overlap, *t*-testing revealed that the target capture contig number distribution was significantly lower than the distribution from Norman et al. (mean = 1774.924 vs. mean = 2850.116, *p* < 2.2e − 16), indicative of more complete assemblies. Correspondingly, the target capture N50 values were significantly larger (mean = 4359.881vs. mean = 3894.989, *p* = 9.584e − 10), indicating larger, median contig length. There was a slight, but significant, decrease in total assembly length in the target capture compared to the Norman et al. sequences (mean = 4,185,033 vs mean = 4,567,339, *p* = 0.0008554). The same trends were observed in the WGS data, though contig number and N50 value had a completely separate distribution from both the target capture and the Norman et al. assemblies (Fig. S10, Additional files 3 and 4).

#### *MHConstructor* assemblies of target capture, ASW-like sequences exhibit different assembly metrics than CEU-like sequences

**Fig. 3:**
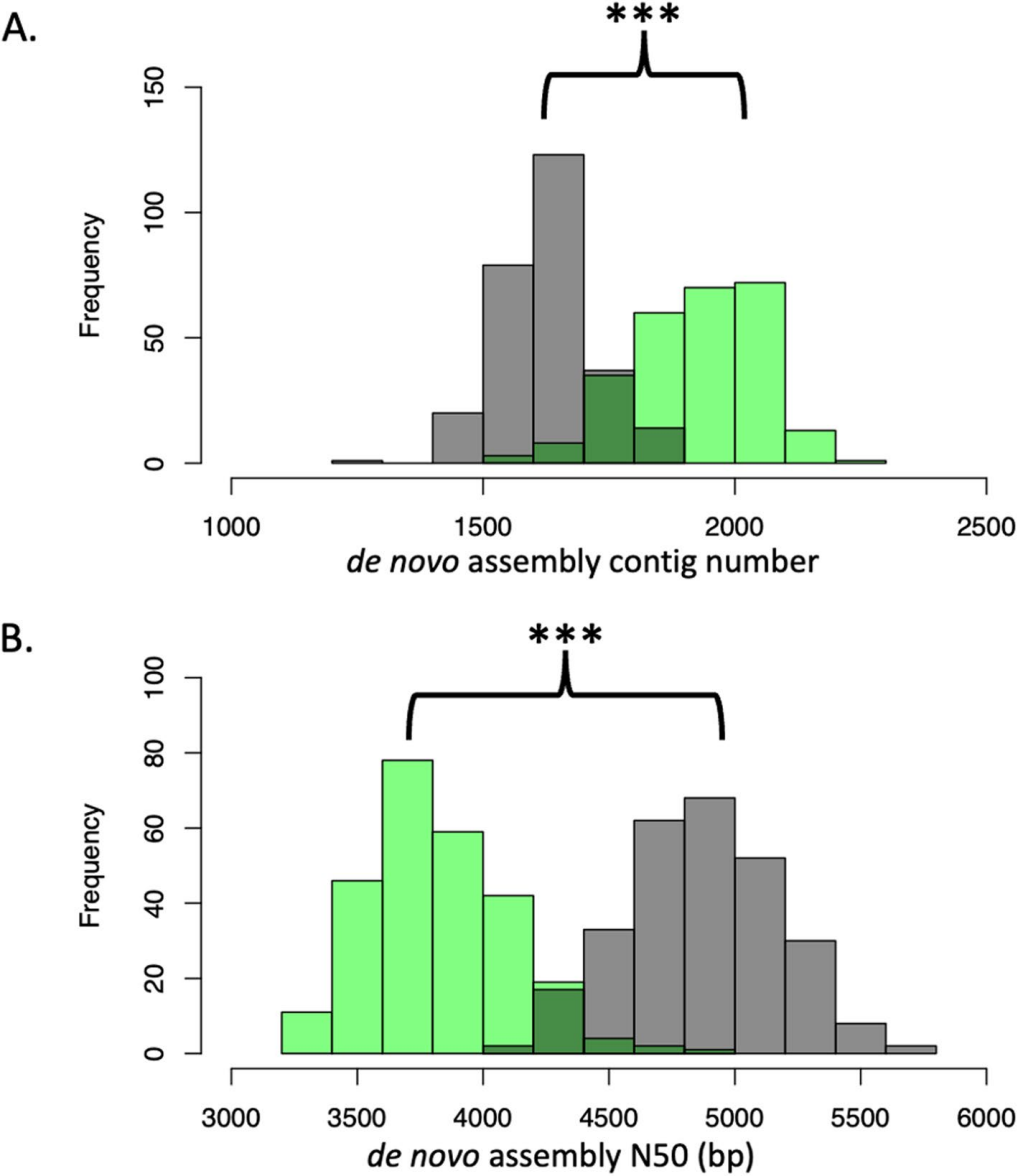
Target capture assemblies from ASW-like individuals demonstrate different assembly metrics than those from CEU-like individuals. Green distributions represent ASW-like assemblies generated from target capture, 60X short-read data, *n* = 262 MHC haplotypes. Grey distributions represent CEU-like assemblies generated from target capture, 60X short-read data, *n* = 274 MHC haplotypes. **A** Histogram of the number of contigs per assembly. **B** Histogram of N50 (bp), per assembly

Interestingly, there is a bimodal distribution within the target capture assembly metric distributions (Fig. 3). The ASW-like assemblies have a higher contig number (*p*-value < 2.2e − 16), lower N50 (*p*-value < 2.2e − 16), and lower total assembly length (*p*-value = 8.673e − 16) than the CEU-like assemblies. Traditionally, these values are associated with lower assembly quality. There was a corresponding difference in terms of kmer diversity between these two populations as well. In the target capture short-read fragments, the mean number of unique 150mers per ASW-like reads was significantly higher than the mean unique 150mers in the CEU-like reads (*p*-value < 2.2e − 16).

Interestingly, when comparing these same assembly metric distributions between the CEU and ASW assemblies generated from 30X 1000 Genomes Project WGS data, no differences were observed in contig number (*p*-value = 0.3184) or N50 length (*p*-value = 0.2629), though total assembly length was slightly higher in the CEU population (*p*-value = 0.004869). Additionally, there was no observable difference in number of unique short-read 150mers between the WGS 1000 Genomes Project short-read fragments between the two populations (*p* = 0.5212) (Fig. S11). To ensure that the increased kmer diversity difference between populations observed in the target capture data were not an artifact of the larger sample size (*n* = 536 haplotypes), relative to the WGS 1000 Genomes Project samples (*n* = 300 haplotypes), we performed random subsampling of the target capture individuals to achieve equivalent sample sizes. Even at a smaller sample size (*n* = 300), there were still significantly more unique 150mers in the target capture ASW-like short-read data (*p*-value < 2.2e − 16).

## Discussion

Though it is known that human MHC variation is implicated in numerous disease phenotypes, aside from the classical HLA loci, characterization of variation in this region has been limited to SNP genotyping arrays, reference-based alignment methods, and, only very recently, long-read sequencing. Because of this, MHC sequences from thousands of disease cohorts with short-read data have remained untapped, and knowledge of the true extent of MHC variation has been obscured. Here, we developed and demonstrated a tool, *MHConstructor* [27], that produces high-resolution, reference-free, de novo short-read assembly of the extended MHC, for both target capture and WGS short-read sequencing methods.

This tool facilitates enhanced MHC research in large human population cohorts in four key areas by (1) applying automated HLA typing and HLA class II haplotype assignment with *T1K* [29], (2) using guide sequences for read binning to improve assembly of HLA class II structural variation and C4 A/B prediction using *C4Investigator* [33], (3) using de novo sequence assembly to capture novel variation, and (4) creating a reproducible *Singularity* container [26] to increase usability.

By comparing *MHConstructor* performance for both target capture and WGS methods, against high-quality, reference MHC haplotypes from long-read sequencing data, we have established an error rate between 0.12% and 0.44% overall, across both types of sequencing technology. We find that target capture assemblies exhibit slightly lower error rates than WGS assemblies. We deemed these acceptable error rates, as the primary use of these sequences will be for the purpose of population-scale, disease association studies. Any spurious variants that occur at or below this frequency threshold will not be expected to demonstrate high enough frequency at the population level to result in false associations, as most statistical models do not have the sensitivity to detect associations for variants with a population frequency < 5%. We demonstrate that the *MHConstructor* haplotype-guided de novo assembly approach enables accurate reconstruction of the known HLA class II structural variation, even in situations where an individual is heterozygous, i.e., has two different HLA class II haplotypes. This finding underscores its applicability to human population datasets and expands its functionality from homozygous cell lines [38]. Furthermore, we also find that *MHConstructor* is sensitive for novel variant discovery, as revealed by our simulation analysis. Novel SNV recovery in diploid simulated sequence was very high (98.0–99.5%). Recovery of novel structural variation in diploid simulated sequence exhibited a slightly wider, but still well-performing range (86.2–99.0%), which is unsurprising, given the challenges of de novo assembly with short-read data [56, 57]. Altogether, this supports the use of *MHConstructor* as a tool for discovery in disease association contexts.

Though overall assembly statistics appear canonically better in the 30X WGS assemblies, the higher error rate as compared to the 65X target capture sequencing implies that these statistics may be falsely inflated. It is possible that the 30X WGS method is not sensitive enough to fully capture the extent of nucleotide diversity across all individuals. This is additionally supported by the comparison of assembly metrics between the two distinct populations considered. In the target capture assemblies, we find “lower” quality scores associated with the assemblies of ASW-like individuals, compared to the CEU-like individuals, suggestive of increased nucleotide diversity in the ASW-like population that may be further challenging the de novo assembly process. This difference is not observed between the 1000 Genomes Project 30X WGS ASW and CEU populations, suggesting that the 65X target capture approach may be picking up more of the nucleotide diversity known to be associated with populations of African American ancestry [58, 59]. We find additional support for this interpretation, given that reads from the target capture sequencing appear capable of identifying a higher amount of likely novel MHC variation in populations with African ancestry than WGS sequencing. It is likely that the lower, 30X coverage depth, commonly used for WGS of large cohorts, may also be contributing to reduced sensitivity. Thus, some novel variation may not be identifiable with a 30X WGS approach, though it is unclear if increasing the WGS coverage depth would account for this. Our results suggest this may have an even greater impact in populations with African ancestry, as opposed to European ancestry. It is likely that we may still be underestimating the true extent of MHC variation.

Due to the large number of assembly software that these processes rely on, the containerized workflow is essential for establishing this method as a usable tool for a wide variety of audiences. Throughout the process of our subsampling and optimization analyses, we find that sequencing depths below 25 × on average across the MHC are not sufficient to generate full assemblies. We find that assemblies at these lower coverage ranges are more fragmented and do not produce full-length consensus sequences. Therefore, we recommend a sequencing depth of 30 × or higher to achieve the most robust assemblies. Higher starting sequencing coverage (45 × and 60x) will generally have higher accuracy but will take longer to complete the assembly. There are many additional parameters involved in this analysis which may require fine-tuning, and we encourage users to consider these with respect to their own project and data. Since every NGS dataset will have unique quality dynamics and experimental questions, we recommend that users perform a similar evaluation as performed in this manuscript when choosing the most relevant parameters for their dataset. We find that, while speed is impacted to some extent by coverage depth and choice of assembly parameters, average runtimes per haplotype range between 1 h to one and a half hours, using eight parallel computing threads at computationally intensive stages. By developing this pipeline structure, executed from within a *Singularity* container, we have made de novo assembly significantly more accessible to a broader range of researchers. Furthermore, this structure ensures that results can be replicated and facilitates additional optimization and improvement due to its modularity, which allows the user to optimize the analysis parameters for each unique dataset, if desired.

An inherent limitation of this method is attributable to the nature of short read sequence data. Since the sequence fragments used in short-read sequencing are generally shorter than 200 bps in length, this can result in assembly errors when the length of a repetitive motif is longer than the length of the read fragment [56, 57]. By comparison to long-read assembled, fully phased MHC sequences, we find that *MHConstructor* introduces an additional 0.086–0.125% error attributable to misassembling interspersed repeats, on average across the MHC. This limitation is noticeably more pronounced at the C4 gene locus. Since C4A and C4B are identical at the nucleotide level, with the exception of four codons, it is almost impossible for de novo assembly algorithms to meaningfully identify the correct path through elements with such a high sequence identity [33]. Similarly, copy number variation is unlikely to be accurately captured from short-read sequence assembly alone and requires an estimation of coverage depth. As evidenced by the dot plot visualization in Fig. 2, showing assembly alignment gaps corresponding to the position of *C4A* and *C4B*, de novo short-read assembly is not capable of discerning between *C4A* and *C4B* and their duplicates. For these reasons, we have implemented the tool *C4Investigator* [33], which is designed to distinguish between the presence of *C4A* and *C4B*, long and short forms, and copy numbers.

Though *MHConstructor* generates first field genotyping of *HLA-DRB1* using *T1K* [29], we do not recommend using this data, or the *MHConstructor* pipeline, for the purposes of clinical HLA genotyping. The primary goal of this analysis is to expand access to large population-level cohorts and foster the discovery of novel variation and factors impacting MHC biology across the extended 5-Mbp region. It is not designed to meet the level of stringency required for assigning HLA genotypes in a clinical context as there are many robust and user-friendly tools and protocols which are designed for that level of required accuracy, and we encourage users to pursue those, should their interest be in clinical HLA typing.

## Conclusions

The modular nature of the pipeline workflow lends itself to adaptions and future modifications. Though it has not been tested within the scope of this manuscript, we anticipate that it should be possible to integrate long read sequencing data with this pipeline as well. This could be introduced at step 3, replacing de novo assembled, short-read contigs. The use of hybrid long and short-read sequencing is well known to be the most effective method of high-accuracy sequence assembly, and including such data here would likely increase assembly accuracy even further. Future work can be done to fine-tune and validate this alternative application. *MHConstructor* will also support the discovery of novel patterns of variation and even never before-described genomic variation. Additional future efforts will likely involve the application of additional methods to enhance the annotation of SVs in the MHC. It is not a trivial task, and algorithms for the characterization of complicated SVs are continuously being improved upon [60]. Future work to formally phase the output of *MHConstructor* will expand its usability even further [61]. Additionally, though it is known that the unique sequence features of the MHC are driven by a combination of nuanced and interacting selective scenarios [62], attempts to model these forces have remained limited in scope. With this new access to extended MHCs from large population cohorts, we can begin to more thoroughly reconstruct those processes, which, in turn, will lead to a better understanding of human disease. In all, the containerized, pipelined workflow established here will make its application to short-read disease-cohort datasets computationally accessible. In this way, *MHConstructor* will encourage functional interpretation of MHC variation and its roles in human disease contexts.

## Methods

### DNA collection for target capture sequencing

DNA from peripheral blood mononuclear cells (PBMCs) of healthy individuals [63, 64] was obtained for target capture sequencing. The study sample for sequencing was comprised of 183 healthy individuals from a population similar to the 1000 Genomes Project Utah residents (CEPH) with Northern and Western European ancestry group and 185 healthy individuals from a population similar to the 1000 Genomes Project African Ancestry in Southwest US group. Populations will be referred to as CEU-like and ASW-like, according to nomenclature recommendations from a National Academies consensus study report [65].

### Target capture sequencing of the extended MHC

For each sample, 100 ng of high-quality DNA was used for library preparation using the Twist Library Preparation EF Kit 2.0 (Twist Bioscience, San Francisco, USA) with enzymatic fragmentation and combinatorial dual indices following manufacturer instructions. Step-by-step instructions for library preparation are provided by the manufacturer’s publicly available protocol. In summary, the first step consists of DNA fragmentation, end-repair, and poly-A tailing (as described in Twist protocol). Subsequently, universal xGen™ stubby adaptors (IDT, Coralville, IA) were ligated using the ligation master mix provided by the Twist Library Preparation EF Kit 2.0 and following the manufacturer’s protocol. Then, fragments were purified with 0.8 × ratio AMPure XP magnetic beads (Beckman Coulter, Brea, USA), followed by dual-size selection (0.42 × and 0.15 × ratios), resulting in libraries of approximately 800 bp. Next, the libraries were amplified with xGen™ UDI 10nt Primers (IDT, Coralville, IA) uniquely barcoded with i5 and i7 index sequences to allow multiplexing. The following amplification settings were used, according to the manufacturer protocol: 1 initialization cycle at 98 °C for 45 s, 8 cycles of denaturation/annealing/extension at 98 °C for 15 s, 60 °C for 30 s and 72 °C for 30 s and a final extension cycle at 72 °C for 1 min. Following amplification, a second round of purification was performed with a 1 × ratio using AMPure XP magnetic beads (Beckman Coulter, Brea, USA), according to manufacturer instructions.

For large-scale quantification, fluorometric quantification was performed using Quant-iT™ PicoGreen™ (Thermofisher, Waltham, USA) following the manufacturer’s instructions and using the Gemini XPS Fluorescence Microplate Reader (Molecular Devices, USA). To automate pooling, samples were pooled (30 ng/sample) via ultrasonic acoustic energy using Echo 525 Liquid Handler (Beckman Coulter, Brea, USA). The Twist Target Enrichment kit (Twist Bioscience, San Francisco, USA) was then used for targeted hybrid capture on pooled libraries. Step-by-step instructions for hybrid capture enrichment are provided by the manufacturer’s publicly available protocol. DNA libraries were bound to 33,620 biotinylated 120-bp probes (Twist Custom Panel, Twist Bioscience, San Francisco, USA) designed by Twist Biosciences targeting the entire extended MHC, including genic and intergenic regions. After overnight incubation, following Twist Hybrid Capture protocol, Twist Dry Down Beads (streptavidin magnetic beads) provided in the kit were used to capture fragments targeted by the probes, according to manufacturer instructions. Captured fragments were then amplified using universal primers provided by the kit and the same amplification setting described above, according to manufacturer instructions. After amplification, libraries were purified with AMPure beads (Beckman Coulter, Brea, USA), according to Twist Hybrid Capture protocol. Bioanalyzer (Agilent, Santa Clara, USA) was then used to analyze the enriched pooled libraries. After quality control evaluation, enriched libraries were sequenced using a paired-end 150-bp sequencing protocol on the NovaSeq 6000 platform (Illumina, San Diego, USA). This protocol is an adaptation of the method previously published by Norman et al. [38]. Short-read source data can be found at the NCBI SRA record SRP487874 [66].

### WGS data collection from the 1000 Genomes Project

The MHC region was extracted from 1000 Genomes Project.cram files corresponding to 30 × WGS data for 214 individuals from the CEU and ASW populations using the following command: $ samtools view -T http://ftp.1000genomes.ebi.ac.uk/vol1/ftp/technical/reference/GRCh38_reference_genome/GRCh38_full_analysis_set_plus_decoy_hla.fa -b -o < sample > .bam < sampleURL > < chromosome > , where < sample > and < sampleURL > correspond to the unique ID and location path on the ftp server for each individual and < chromosome > corresponds to the chromosome region from which to extract reads. Reads were extracted from the canonical MHC region using chr6: 28,509,120–33481577, as well as from the following alternate chromosomes on the hg38 build known to house MHC sequence: chr6_GL000250v2_alt, chr6_KI270800v1_alt, chr6_KI270799v1_alt, chr6_GL383533v1_alt, chr6_KI270801v1_alt, chr6_KI270802v1_alt, chr6_KB021644v2_alt, chr6_KI270797v1_alt, chr6_KI270798v1_alt, chr6_GL000251v2_alt, chr6_GL000252v2_alt, chr6_GL000253v2_alt, chr6_GL000254v2_alt, chr6_GL000255v2_alt, chr6_KI270758v1_alt. Reads which did not map to GRCh38_full_analysis_set_plus_decoy_hla.fa were extracted from each individual’s alignment.bam file using samtools view -u and stored separately for use in later stages of analysis. The full list of 1000 Genomes Project individuals and the ftp links to access corresponding short-read fastq files can be found in Additional file 5.

### Additional MHC-related tools

These processes are separate from the *MHConstructor* pipeline. They include two functions that produce data required for *MHConstructor* to run. However, these modules may be skipped if the user already has these data prepared.

- *HLA-DRB1 genotyping*. In order to assign each individual to the most relevant major MHC haplotype, according to Houwaart et al. [24], we have included a pre-processing step to generate *HLA-DRB1* genotypes at first field resolution and complement component 4 (C4) genotype (C4A or C4B) and copy number. This stage is executed prior to the assembly algorithm. High-throughput *HLA-DRB1* first field genotypes are generated using T1k [29]. Since *HLA-DRB1* is in known to be in strong linkage disequilibrium with *HLA-DRB3*, *HLA-DRB4*, and *HLA-DRB5* [52, 53], we infer that the first field genotypes of *HLA-DRB3*, *HLA-DRB4*, and *HLA-DRB5* will correspond to previously established *HLA-DRB1* haplotypes [24]. In this way, we determine the HLA class II haplotype(s) for each individual.
- *Complement component 4 (C4A and C4B genotyping*. *C4A* and *C4B* genotypes and copy numbers are assigned using C4Investigator [33] with the standard parameter values, calculating C4 copy numbers (C4A, C4B, C4S, C4L).

### *MHConstructor *de novo assembly pipeline

The following describes each functional module involved in *MHConstructor*. Each module is executed by a driver script written in bash, which calls the necessary software to execute the module. The modules associated with read quality filtering, contig generation, and scaffolding are derived from the reference-guided de novo assembly method described by Lischer and Shimizu [43]. For an in-depth description of these methods, please refer to the original publication [43]. Changes made to the original algorithm include replacing the primary assembly software with *velvet* [67] for increased speed, as well as minor code alterations, made to be applicable to human MHC sequence reads and up to date with current software versions.

1. *Short-read quality control*. Fastq files are processed to remove stretches of homopolymer runs from reads, where reads contain a minimum of 20, consecutive guanines (G). Long, consecutive stretches of guanine are a known artifact of Illumina NGS [68]. Then, reads are randomly sampled with a seed value using seqtk (https://github.com/lh3/seqtk) to select the desired number of reads to include in downstream analysis. Sampled reads are then processed with Trimmomatic [69] to remove leading or trailing low-quality bases (< 3 or *N*), remove bases that had an average quality score of 15 or less across four base pair sliding windows, and exclude reads that are shorter than 40 base pairs, according to Lischer and Shimizu [43]. This analysis assumes that sequencing primers have already been removed from the sequencing reads.
2. *MHC BMH assignment and read binning*. Best matching haplotypes (BMHs) are inferred from *HLA*-DRB1 genotyping and C4 data. If an individual is first field homozygous at *HLA*-DRB1, they are assigned a single BMH. If an individual has a heterozygous DR- haplotype, they are assigned two BMHs. Possible recombination was not taken into account in assigning proxy chromosomal haplotypes. Guide BMH sequences were obtained from the following NCBI accession numbers: OK649231.1, OK649232.1, OK649233.1, OK649234.1, OK649235.1, OK649236.1, and the MHC region on chromosome 6 of human reference genome GRCh38.p14, NC_000006.12: chr6: 28,509,120–33481577. Individuals with a DR8 haplotype were assigned GRCh38.p14. Once BMHs are assigned, reads are aligned to each BMH using *bowtie2* [70]. The *bowtie2* minimum alignment score is set to “L,0,-0.6,” according to step 2 of the reference-guided de novo assembly method in Lischer and Shimizu [43]. Reads that align to a given BMH are then extracted for de novo assembly. Reads that do not align to the BMH are extracted and grouped for de novo assembly separate from the reads that aligned. If an individual has two BMHs, the de novo assembly process is conducted separately for each BMH. In these cases, following de novo assembly, contigs generated from reads that did not map to a BMH are later aligned to the heterozygous individual’s alternative BMH and any cross-mapping contigs are removed, as described in the “Quantification of MHConstructor error rates using high-quality MHC reference sequences” section.
3. *Haplotype-informed contig set generation *via* reference-guided, *de novo* assembly*. Non-redundant supercontigs are generated using an individual’s BMH(s) as guide sequence(s) for de novo assembly, according to Lischer and Shimizu [43]. Following alignment to the guide BMH, regions of mapped, overlapping reads are grouped into blocks. These blocks are then merged into “superblocks,” which represent the boundaries of adjacent, overlapping blocks, as defined in Lischer and Shimizu [43]. Reads that do not align to the guide BMH are extracted and assembled separately. Both categories of reads are then extracted and de novo assembled into contigs via de Bruijn kmer graphs, as implemented by *velvet* [67]. *Velvet* was chosen instead of other assemblers described in the original publication [43] for its use of de Bruijn graphs and because its faster speed makes it better suited to high-throughput applications in the context of large disease cohorts. Reads not mapped to the reference sequence are de novo assembled separately. The resulting contigs from the un-mapped sequence are aligned to the rest of the human reference genome (HG38) and, for heterozygous individuals, to the alternative MHC haplotype, using *minimap2* [49]. Any contigs that map outside of the MHC or map to the alternative MHC haplotype reference are removed. This step is an addition to the original algorithm. The remaining filtered contigs generated from unmapped reads are then combined with contigs generated from mapped reads. Finally, overlapping contigs are then merged into non-redundant supercontigs, using *AMOS* [71].
4. *Error-correcting, *de novo* scaffolding, and gap closing of haplotype-informed assemblies*. Original reads are mapped onto the contigs, and *GATK HaplotypeCaller* [72–74] is used to perform localized error-correction. This represents a deviation from the tool described in the original publication [43], as the GATK error correction tool originally chosen is no longer supported by GATK. In the interest of reducing assembly time for use in large cohorts, a secondary round of de novo assembly with the reads which did not map to merged supercontigs was excluded. This can be found in step 5 of the original assembly pipeline [43]. Corrected contigs are then aligned to BMH using *nucmer*, and the resulting delta output files are used as input to *Assemblytics* [50], which establishes contig orientation and identified structural variants. Haplotype-informed contig sets are then processed into draft assemblies, via the Lischer and Shimizu algorithm [43]. *SOAPdenovo2* [75] is used to scaffold error-corrected contigs and close gaps using paired-end mapping to create the haplotype-informed assembly.
5. *Haplotype-informed assembly completion and scaffold orienting*. Draft scaffolds are then ordered and oriented with respect to their major MHC phased reference sequence [24] using RagTag [48]. A final assembly fasta file is created, which contains one single fasta record representing the ordered and continuous sequence assembled with respect to the reference. This serves as a haplotype-informed consensus sequence for each major MHC haplotype. Any de novo assembled contigs that RagTag [48] is not able to place on the BMH guide sequence are output as separate fasta records in the same file as the continuous, consensus sequence. There is currently no way to automatically infer where in the assembly these novel contigs should be placed in a high-throughput manner. Therefore, if users are interested in including the novel contigs, manual, post hoc evaluation and placement of these sequences is required.

### Post hocassembly parameter evaluation and optimization

Following algorithm development, the effect of coverage depth on assembly time, computational cost, and assembly quality was evaluated by randomly subsampling pairs of reads to represent different coverage depths at 200,000 read pairs, 500,000 read pairs, 1 million read pairs, 2 million read pairs, and 3 million read pairs, for each sample. Additionally, to evaluate the most appropriate kmer length for de novo assembly, *KmerGenie* [76] was used to estimate initial ranges of likely successful kmers. This was done separately for the subset of mapped reads and the subset of reads that did not align to the reference. Paired R1 and R2 reads were combined for these estimates. Best kmer predictions were averaged across all samples at a given read count. The average kmer size prediction was then rounded to the closest whole, odd number. Standard deviation of best predicted kmer was also evaluated across all samples. Time, memory usage, and quality were measured separately for super contig and scaffolding stages. Assembly parameters for multithreading, insert size, expected coverage, de novo assembly kmer length, and starting number of reads are listed in the text editable control.txt file included in the *MHConstructor* software package. This file is built into the *Singularity* image and must be edited by the user to be appropriate for each individual analysis.

### *MHConstructor* validation using high-quality MHC reference sequences

To validate the accuracy of assemblies generated with this method, we applied it to independently generated Illumina short-read datasets, corresponding to the MHC region of HLA-homozygous, consanguineous cell lines (*SRA:* SRP348947, *BioProject:* PRJNA764575) [38]. We chose five cell lines that were also thoroughly assembled and curated in a later study via a combination approach using both short and long-read sequencing technology [24]. These high-quality findings regarding the impact of sequencing finished sequences were set to represent “ground truth.” The extent to which de novo assembly with *MHConstructor* was able to reproduce these ground truth sequences from the same source data was used to evaluate both *MHConstructor*’s accuracy and its errors as well as the types of sequences that are driving its error. Following assembly of these five short-read datasets using our own de novo approach, we aligned our new assemblies to the curated, full-length MHC phased sequence for that same cell line, acting as the gold standard. Any variation with respect to the reference sequence was counted as an error. Base pair accuracy validation was performed using *minimap2* to align *MHConstructor* sequences to the corresponding reference sequence and calling SNPs using *paftools*. Structural errors ≥ 2 bp INDELs were identified using the assembly-based variant predictor *Assemblytics* [50]. The nucleotide length of each error was summed for each size category, and a final percent error was calculated for each assembly by dividing the number of erroneous base pairs in each category by the full length of assembled sequence. To evaluate the impact of heterozygous, diploid sequence data on assembly accuracy, we used three 1000 Genomes Project 30X WGS samples with long read data that has recently been assembled into phased MHC haplotypes [47].

### *MHConstructor *validation via simulation

To evaluate the performance of *MHConstructor* with respect to ground truth MHC haplotype sequences, we generated simulated short-read fragments from phased MHC haplotype sequences [47]. We used the *ART* simulation software, version “MountRainer” [51], with simulation settings chosen such that simulated reads would exhibit similar characteristics as those generated with the WGS probes used in the 1000 Genomes Project sequencing initiative: art_illumina -ss HSXt -sam -p -l 150 -f 30 -m 426 -s 109. Synthetic reads were then assembled with *MHConstructor*. We evaluated the sensitivity of *MHConstructor* to identify novel variation, not found in the guide BMH, by identifying the set of nucleotide variants which were unique to the ground truth haplotype sequences, i.e., not present in the guide BMH sequence. These sites representing novel variation were then evaluated for correct annotation in the *MHConstructor* assemblies of the synthetic, simulated reads.

### Repeat annotation and masking

To characterize the repetitive elements located within novel MHC assemblies, we applied repeatMasker v4 (http://www.repeatmasker.org) to the consensus sequence(s) from each assembly, using default parameters. Following characterization, repeat regions were then masked. Masking is the process of replacing the nucleotide sequence of regions identified to be repetitive elements with “*N*,” in place of each nucleotide base. This was also carried out with repeatMasker using default settings.

## Supplementary Information

Additional file 1. 1st field HLA-DRB1 genotypes for the individuals in this study, generated by *T1k* [29] from custom, target-capture short-read sequencing.

Additional file 2. C4A/B and C4S/L genotyping and copy numbers for the individuals in this study, generated from custom, target capture short-read sequencing. C4 genotyping performed by *C4Investigator* [33].

Additional file 3. Final *MHConstructor* MHC assembly metrics generated from target-capture short-read sequencing reads.

Additional file 4. Final *MHConstructor* MHC assembly metrics, generated from the 1000 Genomes Project 30X WGS short-read sequencing data for the ASW and CEU populations.

Additional file 5. FTP links to 30X WGS alignment files (.cram) for individuals from the 1000 Genomes Project ASW and CEU populations that were used in this study. These can be used to access the 1000 Genomes Project source reads.

Additional file 6. Review history.

Additional file 7.

Additional file 8.

## Data Availability

The *MHConstructor* [27] software is publicly available at www.github.com/Hollenbach-lab/MHConstructor, https://doi.org/10.5281/zenodo.13763874. Target capture sequencing reads from healthy individuals were generated in affiliation with dbGap study phs003244.v1.p1 and source data can be accessed there [66]. Additional intermediate data generated throughout the de novo assembly process is available upon request.

## References

[1] Boyle EA, Li YI, Pritchard JK. An expanded view of complex traits: from polygenic to omnigenic. Cell. 2017;169:1177–86. 10.1016/j.cell.2017.05.038. 28622505 10.1016/j.cell.2017.05.038 PMC5536862

[2] Lenz TL, Spirin V, Jordan DM, Sunyaev SR. Excess of deleterious mutations around HLA genes reveals evolutionary cost of balancing selection. Mol Biol Evol. 2016;33:2555–64. 10.1093/MOLBEV/MSW127. 27436009 10.1093/molbev/msw127 PMC5026253

[3] Aguilar A, Roemer G, Debenham S, Binns M, Garcelon D, et al. High MHC diversity maintained by balancing selection in an otherwise genetically monomorphic mammal. Proc Natl Acad Sci U S A. 2004;101:3490–4. 10.1073/PNAS.0306582101/SUPPL_FILE/06582TABLE4.HTML. 14990802 10.1073/pnas.0306582101 PMC373489

[4] Sommer S. The importance of immune gene variability (MHC) in evolutionary ecology and conservation. Front Zool. 2005;2: 16. 10.1186/1742-9994-2-16. 16242022 10.1186/1742-9994-2-16 PMC1282567

[5] Trowsdale J, Knight JC. Major histocompatibility complex genomics and human disease. Annu Rev Genomics Hum Genet. 2013;14:301. 10.1146/ANNUREV-GENOM-091212-153455. 23875801 10.1146/annurev-genom-091212-153455 PMC4426292

[6] Doherty PC, Zinkernagel RM. Enhanced immunological surveillance in mice heterozygous at the H-2 gene complex. Nature. 1975;256:50–2. 10.1038/256050a0. 1079575 10.1038/256050a0

[7] Hudson RR, Kaplan NL. The coalescent process in models with selection and recombination. Genetics. 1988;120:831–40. 10.1093/genetics/120.3.831. 3147214 10.1093/genetics/120.3.831 PMC1203560

[8] Kaufman J. Unfinished business: evolution of the MHC and the adaptive immune system of jawed vertebrates. Annu Rev Immunol. 2018;36:383–409. 10.1146/annurev-immunol-051116-052450. 29677478 10.1146/annurev-immunol-051116-052450

[9] Radwan J, Babik W, Kaufman J, Lenz TL, Winternitz J. Advances in the evolutionary understanding of MHC polymorphism. Trends Genet. 2020;36:298–311. 10.1016/J.TIG.2020.01.008. 32044115 10.1016/j.tig.2020.01.008

[10] Robinson J, Guethlein LA, Cereb N, Yang SY, Norman PJ, et al. Distinguishing functional polymorphism from random variation in the sequences of >10,000 HLA-A, -B and -C alleles. PLoS Genet. 2017;13: e1006862. 10.1371/journal.pgen.1006862. 28650991 10.1371/journal.pgen.1006862 PMC5507469

[11] Spurgin LG, Richardson DS. How pathogens drive genetic diversity: MHC, mechanisms and misunderstandings. Proc Biol Sci. 2010;277:979–88. 10.1098/rspb.2009.2084. 20071384 10.1098/rspb.2009.2084 PMC2842774

[12] Takahata N, Satta Y. Footprints of intragenic recombination at HLA loci. Immunogenetics. 1998;47:430–41. 10.1007/s002510050380. 9553149 10.1007/s002510050380

[13] Talarico L, Marta S, Rossi AR, Crescenzo S, Petrosino G, et al. Balancing selection, genetic drift, and human-mediated introgression interplay to shape MHC (functional) diversity in Mediterranean brown trout. Ecol Evol. 2021;11:10026–41. 10.1002/ece3.7760. 34367556 10.1002/ece3.7760 PMC8328470

[14] Wakeland EK, Boehme S, She JX, Lu CC, McIndoe RA, et al. Ancestral polymorphisms of MHC class II genes: divergent allele advantage. Immunol Res. 1990;9:115–22. 10.1007/BF02918202. 2189934 10.1007/BF02918202

[15] Beecham AH, Patsopoulos NA, Xifara DK, Davis MF, Kemppinen A, et al. Analysis of immune-related loci identifies 48 new susceptibility variants for multiple sclerosis. Nat Genet. 2013;45:1353–62. 10.1038/NG.2770. 24076602 10.1038/ng.2770 PMC3832895

[16] Beecham AH, Amezcua L, Chinea A, Manrique CP, Gomez L, et al. Ancestral risk modification for multiple sclerosis susceptibility detected across the major histocompatibility complex in a multi-ethnic population. PLoS One. 2022;17: e0279132. 10.1371/journal.pone.0279132. 36548255 10.1371/journal.pone.0279132 PMC9778564

[17] Hollenbach JA, Oksenberg JR. The immunogenetics of multiple sclerosis: a comprehensive review. J Autoimmun. 2015;64:13. 10.1016/J.JAUT.2015.06.010. 26142251 10.1016/j.jaut.2015.06.010 PMC4687745

[18] Matzaraki V, Kumar V, Wijmenga C, Zhernakova A. 2017 The MHC locus and genetic susceptibility to autoimmune and infectious diseases. Genome Biol. 2017;18:1–21. 10.1186/S13059-017-1207-1. 28449694 10.1186/s13059-017-1207-1 PMC5406920

[19] International MHC and Autoimmunity Genetics Network (IMAGEN), Rioux JD, Goyette P, Vyse TJ, Hammarström L, et al. Mapping of multiple susceptibility variants within the MHC region for 7 immune-mediated diseases. Proc Natl Acad Sci. 2009;106:18680–5. 10.1073/pnas.0909307106. 19846760 10.1073/pnas.0909307106 PMC2773992

[20] Morris DL, Taylor KE, Fernando MMA, Nititham J, Alarcón-Riquelme ME, et al. Unraveling multiple MHC gene associations with systemic lupus erythematosus: model choice indicates a role for HLA alleles and non-HLA genes in Europeans. The American Journal of Human Genetics. 2012;91:778–93. 10.1016/j.ajhg.2012.08.026. 23084292 10.1016/j.ajhg.2012.08.026 PMC3487133

[21] Patsopoulos NA, Barcellos LF, Hintzen RQ, Schaefer C, van Duijn CM, et al. Fine-mapping the genetic association of the major histocompatibility complex in multiple sclerosis: HLA and non-HLA effects. PLoS Genet. 2013;9: e1003926. 10.1371/journal.pgen.1003926. 24278027 10.1371/journal.pgen.1003926 PMC3836799

[22] Patsopoulos NA, Baranzini SE, Santaniello A, Shoostari P, Cotsapas C, et al. Multiple sclerosis genomic map implicates peripheral immune cells and microglia in susceptibility. Science. 2019;365: eaav7188. 10.1126/science.aav7188. 31604244 10.1126/science.aav7188 PMC7241648

[23] Dilthey AT. State-of-the-art genome inference in the human MHC. Int J Biochem Cell Biol. 2021;131: 105882. 10.1016/J.BIOCEL.2020.105882. 33189874 10.1016/j.biocel.2020.105882

[24] Houwaart T, Scholz S, Pollock N, Palmer W, Kichula K, Strelow D, et al. Complete sequences of six Major Histocompatibility Complex haplotypes, including all the major MHC class II structure. 2022. 10.1101/2022.04.28.489875. 10.1111/tan.15020 PMC10986641 36932816

[25] Rhie A, McCarthy SA, Fedrigo O, Damas J, Formenti G, et al. Towards complete and error-free genome assemblies of all vertebrate species. Nature. 2021;592:737–46. 10.1038/s41586-021-03451-0. 33911273 10.1038/s41586-021-03451-0 PMC8081667

[26] Kurtzer GM, Sochat V, Bauer MW. Singularity: scientific containers for mobility of compute. PLoS One. 2017;12: e0177459. 10.1371/journal.pone.0177459. 28494014 10.1371/journal.pone.0177459 PMC5426675

[27] Wade KJ, Suseno R, Kizer K, Williams J, Boquett J, Callier S, Pollock NR, Renschen A, Santaniello A, Oksenberg JR, Norman PJ, Augusto DG, Hollenbach JA. MHConstructor (Version 1.0) Github. 2024. 10.5281/zenodo.13763874. 10.1186/s13059-024-03412-6 PMC11484429 39420419

[28] Wang S, Wang M, Chen L, Pan G, Wang Y, et al. SpecHLA enables full-resolution HLA typing from sequencing data. Cell Reports Methods. 2023;3: 100589. 10.1016/j.crmeth.2023.100589. 37714157 10.1016/j.crmeth.2023.100589 PMC10545945

[29] Song L, Bai G, Liu XS, Li B, Li H. Efficient and accurate KIR and HLA genotyping with massively parallel sequencing data. Genome Res. 2023;33:923–31. 10.1101/gr.277585.122. 37169596 10.1101/gr.277585.122 PMC10519407

[30] Cameron DL, Di Stefano L, Papenfuss AT. Comprehensive evaluation and characterisation of short read general-purpose structural variant calling software. Nat Commun. 2019;10:3240. 10.1038/s41467-019-11146-4. 31324872 10.1038/s41467-019-11146-4 PMC6642177

[31] Jensen JM, Villesen P, Friborg RM, Mailund T, et al. Assembly and analysis of 100 full MHC haplotypes from the Danish population. Genome Res. 2017;27:1597–607. 10.1101/gr.218891.116. 28774965 10.1101/gr.218891.116 PMC5580718

[32] Koren S, Rhie A, Walenz BP, Dilthey AT, Bickhart DM, et al. De novo assembly of haplotype-resolved genomes with trio binning. Nat Biotechnol. 2018. 10.1038/nbt.4277.10.1038/nbt.4277. 10.1038/nbt.4277 PMC6476705 30346939

[33] Marin WM, Augusto DG, Wade KJ, Hollenbach JA. High-throughput complement component 4 genomic sequence analysis with C4Investigator. HLA. 2024;103: e15273. 10.1111/tan.15273. 37899688 10.1111/tan.15273 PMC11099535

[34] Pierini F, Lenz TL. Divergent allele advantage at human MHC genes: signatures of past and ongoing selection. Mol Biol Evol. 2018;35:2145–58. 10.1093/molbev/msy116. 29893875 10.1093/molbev/msy116 PMC6106954

[35] Trowsdale J. The MHC, disease and selection. Immunol Lett. 2011;137:1–8. 10.1016/j.imlet.2011.01.002. 21262263 10.1016/j.imlet.2011.01.002

[36] Auton A, Abecasis GR, Altshuler DM, Durbin RM, Abecasis GR, et al. A global reference for human genetic variation. Nature. 2015;526:68–74. 10.1038/nature15393. 26432245 10.1038/nature15393 PMC4750478

[37] Maretty L, Jensen JM, Petersen B, Sibbesen JA, Liu S, et al. Sequencing and de novo assembly of 150 genomes from Denmark as a population reference. Nature. 2017;548:87–91. 10.1038/nature23264. 28746312 10.1038/nature23264

[38] Norman PJ, Norberg SJ, Guethlein LA, Nemat-Gorgani N, Royce T, et al. Sequences of 95 human MHC haplotypes reveal extreme coding variation in genes other than highly polymorphic HLA class I and II. Genome Res. 2017;27:813–23. 10.1101/gr.213538.116. 28360230 10.1101/gr.213538.116 PMC5411776

[39] Miller JR, Koren S, Sutton G. Assembly algorithms for next-generation sequencing data. Genomics. 2010;95:315–27. 10.1016/j.ygeno.2010.03.001. 20211242 10.1016/j.ygeno.2010.03.001 PMC2874646

[40] Nagarajan N, Pop M. Sequence assembly demystified. Nat Rev Genet. 2013;14:157–67. 10.1038/nrg3367. 23358380 10.1038/nrg3367

[41] Besenbacher S, Liu S, Izarzugaza JMG, Grove J, Belling K, et al. Novel variation and de novo mutation rates in population-wide de novo assembled Danish trios. Nat Commun. 2015;6:5969. 10.1038/ncomms6969. 25597990 10.1038/ncomms6969 PMC4309431

[42] Khan AR, Pervez MT, Babar ME, Naveed N, Shoaib M. A comprehensive study of de novo genome assemblers: current challenges and future prospective. Evol Bioinform Online. 2018;14: 1176934318758650. 10.1177/1176934318758650. 29511353 10.1177/1176934318758650 PMC5826002

[43] Lischer HEL, Shimizu KK. Reference-guided de novo assembly approach improves genome reconstruction for related species. BMC Bioinformatics. 2017;18:1–12. 10.1186/S12859-017-1911-6/FIGURES/6. 29126390 10.1186/s12859-017-1911-6 PMC5681816

[44] Baker M. 1,500 scientists lift the lid on reproducibility. Nature. 2016;533:452–4. 10.1038/533452a. 27225100 10.1038/533452a

[45] Cohen-Boulakia S, Belhajjame K, Collin O, Chopard J, Froidevaux C, et al. Scientific workflows for computational reproducibility in the life sciences: status, challenges and opportunities. Futur Gener Comput Syst. 2017;75:284–98. 10.1016/j.future.2017.01.012.

[46] Cokelaer T, Cohen-Boulakia S, Lemoine F. Reprohackathons: promoting reproducibility in bioinformatics through training. Bioinformatics. 2023;39:i11–20. 10.1093/bioinformatics/btad227. 37387150 10.1093/bioinformatics/btad227 PMC10311340

[47] Huijse L, Adams SM, Burton JN, David JK, Julian RS, et al. A pan-MHC reference graph with 246 fully contiguous phased sequences. bioRxiv 2023.09.01.555813. 10.1101/2023.09.01.555813.

[48] Alonge M, Lebeigle L, Kirsche M, Jenike K, Ou S, et al. Automated assembly scaffolding using RagTag elevates a new tomato system for high-throughput genome editing. Genome Biol. 2022;23:258. 10.1186/s13059-022-02823-7. 36522651 10.1186/s13059-022-02823-7 PMC9753292

[49] Li H. Minimap2: pairwise alignment for nucleotide sequences. Bioinformatics. 2018;34:3094–100. 10.1093/bioinformatics/bty191. 29750242 10.1093/bioinformatics/bty191 PMC6137996

[50] Nattestad M, Schatz MC. Assemblytics: a web analytics tool for the detection of variants from an assembly. Bioinformatics. 2016;32:3021–3. 10.1093/bioinformatics/btw369. 27318204 10.1093/bioinformatics/btw369 PMC6191160

[51] Huang W, Li L, Myers JR, Marth GT. ART: a next-generation sequencing read simulator. Bioinformatics. 2012;28:593–4. 10.1093/bioinformatics/btr708. 22199392 10.1093/bioinformatics/btr708 PMC3278762

[52] Maiers M, Gragert L, Klitz W. High-resolution HLA alleles and haplotypes in the United States population. Hum Immunol. 2007;68:779–88. 10.1016/J.HUMIMM.2007.04.005. 17869653 10.1016/j.humimm.2007.04.005

[53] Rollini P, Mach B, Gorski J. Linkage map of three HLA-DR beta-chain genes: evidence for a recent duplication event. Proc Natl Acad Sci U S A. 1985;82:7197–201. 10.1073/pnas.82.21.7197. 3933002 10.1073/pnas.82.21.7197 PMC390816

[54] Darling ACE, Mau B, Blattner FR, Perna NT. Mauve: multiple alignment of conserved genomic sequence with rearrangements. Genome Res. 2004;14:1394–403. 10.1101/gr.2289704. 15231754 10.1101/gr.2289704 PMC442156

[55] de Koning APJ, Gu W, Castoe TA, Batzer MA, Pollock DD. Repetitive elements may comprise over two-thirds of the human genome. Copenhaver GP, editor. PLoS Genet. 2011;7(12):e1002384. 10.1371/journal.pgen.1002384. 10.1371/journal.pgen.1002384 PMC3228813 22144907

[56] Alkan C, Sajjadian S, Eichler EE. Limitations of next-generation genome sequence assembly. Nat Methods. 2011;8:61–5. 10.1038/nmeth.1527. 21102452 10.1038/nmeth.1527 PMC3115693

[57] Dida F, Yi G. Empirical evaluation of methods for de novo genome assembly. PeerJ Comput Sci. 2021;7: e636. 10.7717/peerj-cs.636. 10.7717/peerj-cs.636 PMC8279138 34307867

[58] Campbell MC, Tishkoff SA. African genetic diversity: implications for human demographic history, modern human origins, and complex disease mapping. Annu Rev Genomics Hum Genet. 2008;9:403–33. 10.1146/annurev.genom.9.081307.164258. 18593304 10.1146/annurev.genom.9.081307.164258 PMC2953791

[59] Tucci S, Akey JM. The long walk to African genomics. Genome Biol. 2019;20:130. 10.1186/s13059-019-1740-1. 31248437 10.1186/s13059-019-1740-1 PMC6598360

[60] Kosugi S, Kamatani Y, Harada K, Tomizuka K, Momozawa Y, et al. Detection of trait-associated structural variations using short-read sequencing. Cell Genomics. 2023;3: 100328. 10.1016/j.xgen.2023.100328. 37388916 10.1016/j.xgen.2023.100328 PMC10300613

[61] Kajitani R, Yoshimura D, Okuno M, Minakuchi Y, Kagoshima H, et al. Platanus-allee is a de novo haplotype assembler enabling a comprehensive access to divergent heterozygous regions. Nat Commun. 2019;10:1702. 10.1038/s41467-019-09575-2. 30979905 10.1038/s41467-019-09575-2 PMC6461651

[62] Hedrick PW. Balancing selection and MHC. Genetica. 1998;104:207–14. 10.1023/a:1026494212540. 10386384 10.1023/a:1026494212540

[63] Hollenbach JA, Norman PJ, Creary LE, Damotte V, Montero-Martin G, et al. A specific amino acid motif of HLA-DRB1 mediates risk and interacts with smoking history in Parkinson’s disease. Proc Natl Acad Sci U SA. 2019;116:7419–24. 10.1073/PNAS.1821778116. 10.1073/pnas.1821778116 PMC6462083 30910980

[64] Oksenberg JR, Barcellos LF, Cree BAC, Baranzini SE, Bugawan TL, et al. Mapping multiple sclerosis susceptibility to the HLA-DR locus in African Americans. Am J Hum Genet. 2004;74:160–7. 10.1086/380997. 14669136 10.1086/380997 PMC1181903

[65] National Academies of Sciences, Engineering, and Medicine; Division of Behavioral and Social Sciences and Education; Health and Medicine Division; Committee on Population; Board on Health Sciences Policy; Committee on the Use of Race, Ethnicity, and Ancestry as Population Descriptors in Genomics Research. Using population descriptors in genetics and genomics research: a new framework for an evolving field. Washington (DC): National Academies Press (US); 2023. 36989389

[66] Wade KJ, Suseno R, Kizer K, Williams J, Boquett J, Callier S, Pollock NR, Renschen A, Santaniello A, Oksenberg JR, Norman PJ, Augusto DG, Hollenbach JA. MHConstructor. Target capture Illumina short reads. Sequence read archives (SRA). 2024. https://www.ncbi.nlm.nih.gov/Traces/study/?acc=SRP487874&o=acc_s%3Aa.

[67] Zerbino DR, Birney E. Velvet: algorithms for de novo short read assembly using de Bruijn graphs. Genome Res. 2008;18:821–9. 10.1101/gr.074492.107. 18349386 10.1101/gr.074492.107 PMC2336801

[68] Stoler N, Nekrutenko A. Sequencing error profiles of Illumina sequencing instruments. NAR Genom Bioinform. 2021;3:lqab019. 10.1093/nargab/lqab019. 10.1093/nargab/lqab019 PMC8002175 33817639

[69] Bolger AM, Lohse M, Usadel B. Trimmomatic: a flexible trimmer for Illumina sequence data. Bioinformatics. 2014;30:2114–20. 10.1093/bioinformatics/btu170. 24695404 10.1093/bioinformatics/btu170 PMC4103590

[70] Langmead B, Salzberg SL. Fast gapped-read alignment with Bowtie 2. Nat Methods. 2012;9:357–9. 10.1038/nmeth.1923. 22388286 10.1038/nmeth.1923 PMC3322381

[71] Treangen TJ, Sommer DD, Angly FE, Koren S, Pop M. Next generation sequence assembly with AMOS. Curr Protoc Bioinformatics. 2011;33(1):11–8. 10.1002/0471250953.bi1108s33. 10.1002/0471250953.bi1108s33 PMC3072823 21400694

[72] DePristo MA, Banks E, Poplin R, Garimella KV, Maguire JR, et al. A framework for variation discovery and genotyping using next-generation DNA sequencing data. Nat Genet. 2011;43:491–8. 10.1038/ng.806. 21478889 10.1038/ng.806 PMC3083463

[73] McKenna A, Hanna M, Banks E, Sivachenko A, Cibulskis K, et al. The genome analysis toolkit: a MapReduce framework for analyzing next-generation DNA sequencing data. Genome Res. 2010;20:1297–303. 10.1101/gr.107524.110. 20644199 10.1101/gr.107524.110 PMC2928508

[74] Van der Auwera GA, Carneiro MO, Hartl C, Poplin R, Del Angel G, et al. From FastQ data to high confidence variant calls: the genome analysis toolkit best practices pipeline. Curr Protoc Bioinformatics. 2013;43:11.10.1-11.10.33. 10.1002/0471250953.bi1110s43. 10.1002/0471250953.bi1110s43 PMC4243306 25431634

[75] Luo R, Liu B, Xie Y, Li Z, Huang W, et al. SOAPdenovo2: an empirically improved memory-efficient short-read de novo assembler. Gigascience. 2012;1: 18. 10.1186/2047-217X-1-18. 23587118 10.1186/2047-217X-1-18 PMC3626529

[76] Chikhi R, Medvedev P. Informed and automated k-mer size selection for genome assembly. Bioinformatics. 2014;30:31–7. 10.1093/bioinformatics/btt310. 23732276 10.1093/bioinformatics/btt310

