# Supplementary material for: MHConstructor: a high-throughput, haplotype-informed solution to the MHC assembly challenge
Source: Genome Biol. 2024 Oct 17;25:274. doi: 10.1186/s13059-024-03412-6 (PMC11484429; doi:10.1186/s13059-024-03412-6)
Supplement: Supplementary file 8 — Additional file 8. [file 13059_2024_3412_MOESM8_ESM.pdf]

Supplemental Figures

Table of contents:

Results

1. Haplotype-guided, *de novo* short read MHC assembly workflow

2. Evaluation of assembly performance metrics

Fig S1. Impact of kmer size choice on *de novo* assembly contig metrics, for mapped read k= 41, k=51 and k=61.

Fig S2. Impact of starting read count on *de novo* assembly contig metrics.

3. Quantification of MHConstructor error rates using high-quality MHC reference sequences

Fig S3. Dotplot of *Assemblytics* assembly alignments between 1KGP WGS MHConstructor assemblies (y-axes) and their corresponding, phased reference MHC haplotypes (x-axes).

Fig S4. *Assemblytics* analysis of false SV composition for the MHConstructor *de novo* assembly of HG00621, as compared to phased, reference MHC haplotypes for HG00621.

Fig S5. *Assemblytics* analysis of false SV composition for the MHConstructor *de novo* assembly of NA19240, as compared to phased, reference MHC haplotypes for NA19240.

Fig S6. *Assemblytics* analysis of false SV composition for the MHConstructor *de novo* assembly of HG00621, as compared to phased, reference MHC haplotypes for NA20129.

Fig S7. Mauve alignment plots confirm that the two DR haplotypes for heterozygous Individual Z are correctly assembled.

Fig S8. HLA Class II structure can be correctly assembled even when an incorrectly-match haplotype is used as the assembly guide, so long as the correct haplotype is used for scaffolding orientation.

4. MHConstructor assembly of target-capture 60x and 1000genomes 30x WGS short read MHC data.

Fig S9. MHConstructor assembly metrics for target capture and WGS assemblies.

Fig S10. Nucleotide diversity within sequencing reads.

A.

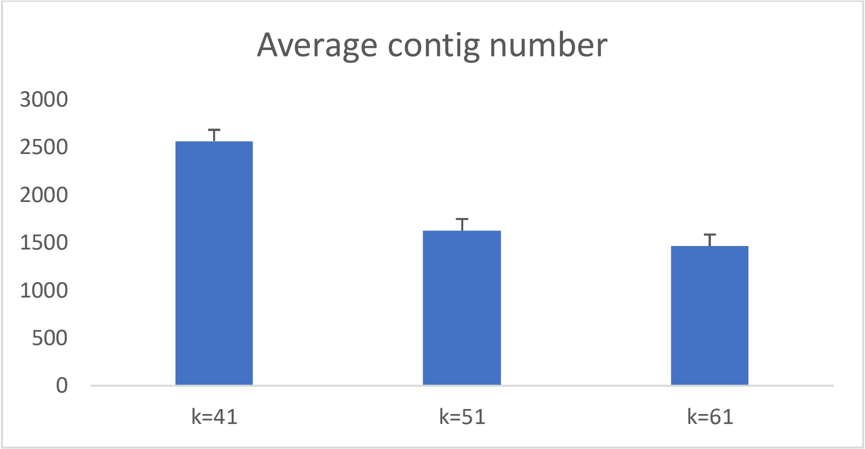

B.

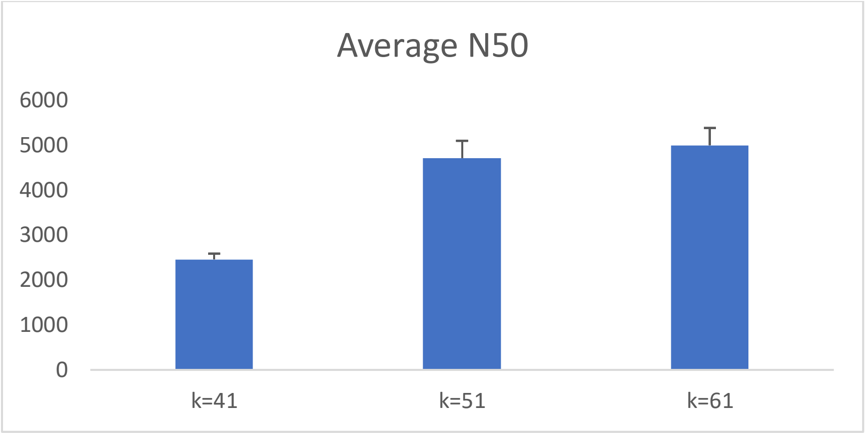

C.

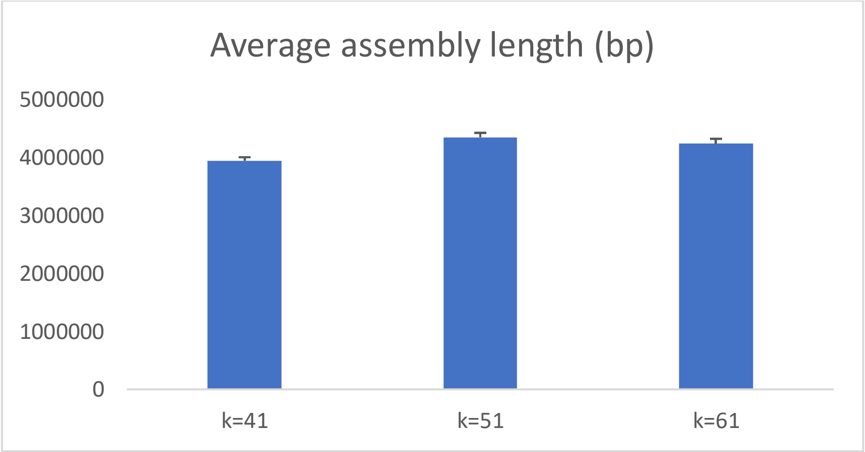

Fig S1. Impact of kmer size choice on *de novo* assembly contig metrics, for mapped read k= 41, k=51 and k=61. Test samples subsampled to contain 2 million reads per individual, n=14. A) Average number of contigs generated for each set of subsampled reads. B) Average N50 length (in bp) for each set of subsampled reads. C) Average length (bp) of contigs generated by *de novo* assembly

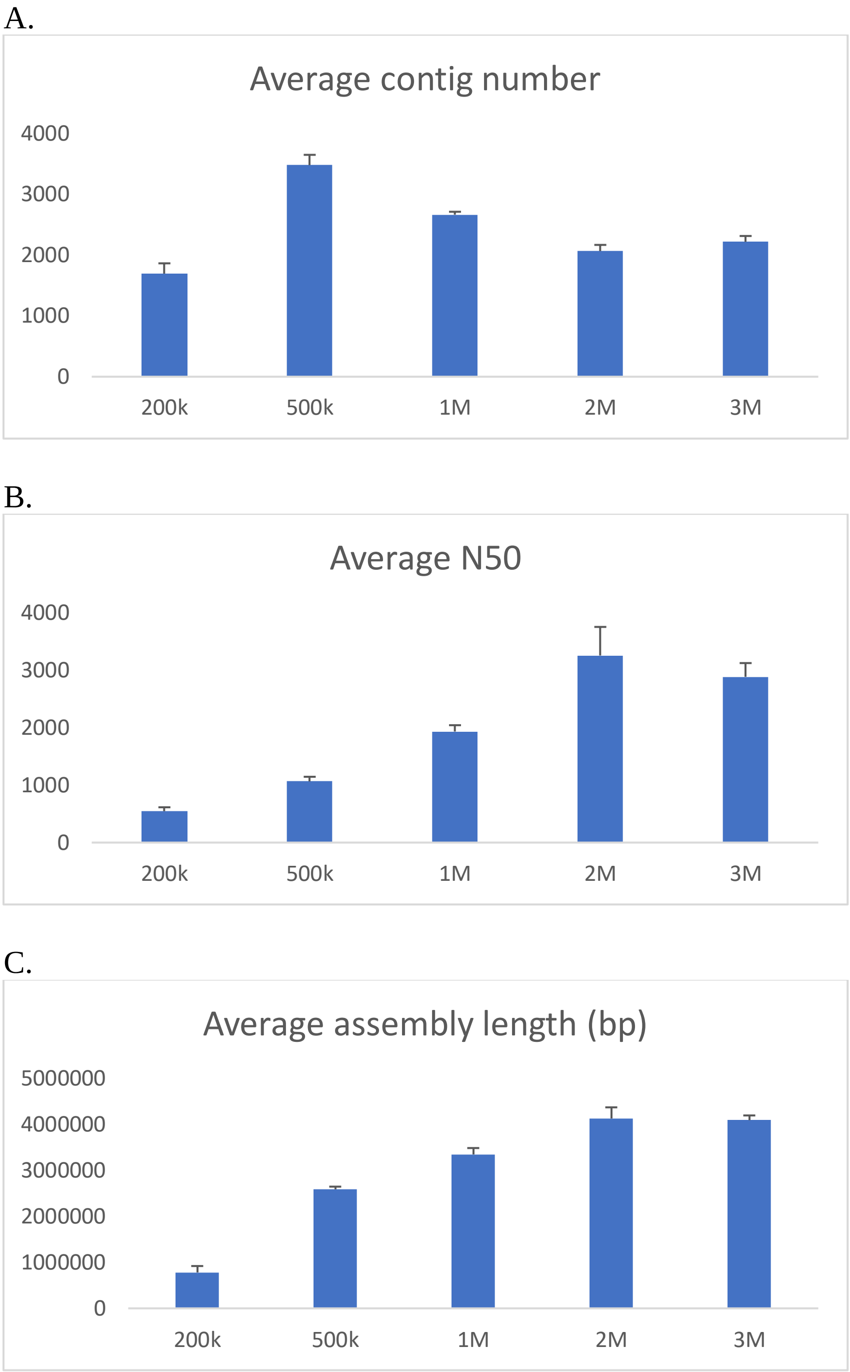

Fig S2. Impact of starting read count on *De novo* assembly contig metrics. Metrics were generated for a test dataset (n=5) of randomly sampled, starting read counts: 200,000 (200k), 500,000 (500k), 1 million (1M), 2 million (2M) and 3 million (3M). A) Average number of contigs generated for each set of subsampled reads. B) Average N50 length (in bp) for each set of subsampled reads. C) Average length (bp) of contigs generated by *de novo* assembly.

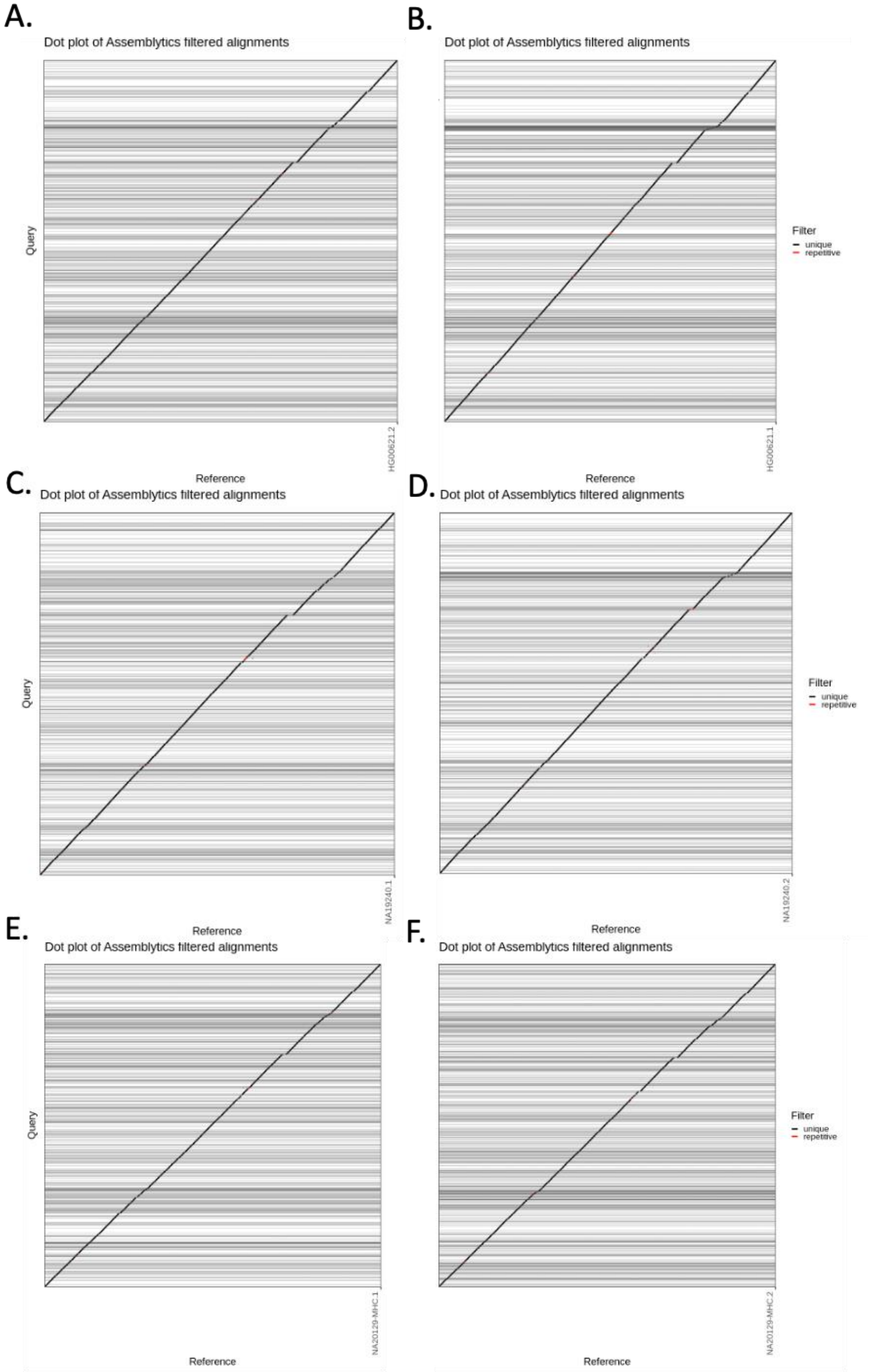

Fig S3. Dotplot of *Assemblytics* assembly alignments between 1KGP WGS MHConstructor assemblies (y-axes) and their corresponding, phased reference MHC haplotypes (x-axes). The assemblies shown represent: HG00621 MHC haplotype A (A), HG00621 MHC haplotype B (B), NA19240 MHC haplotype A (C), NA19240 MHC haplotype B (D), NA20129 MHC haplotype A (E), NA20129 MHC haplotype B (F).

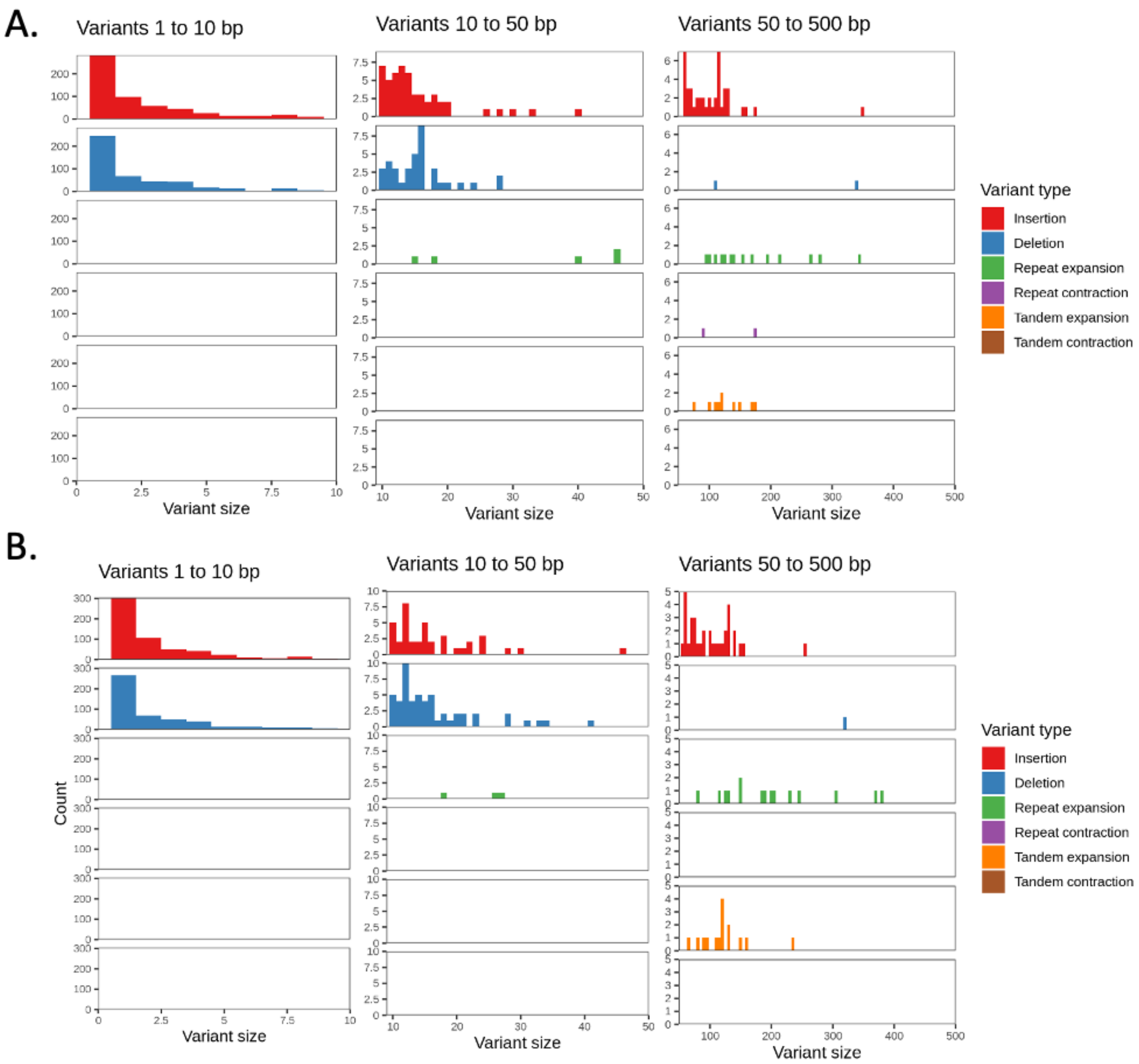

Fig S4. *Assemblytics* analysis of false SV composition for the MHConstructor *de novo* assembly of HG00621, as compared to phased, reference MHC haplotypes for HG00621. A) HG00621 MHC haplotype A, B) HG00621 MHC haplotype B. Categories of assembly SV errors described in legend.

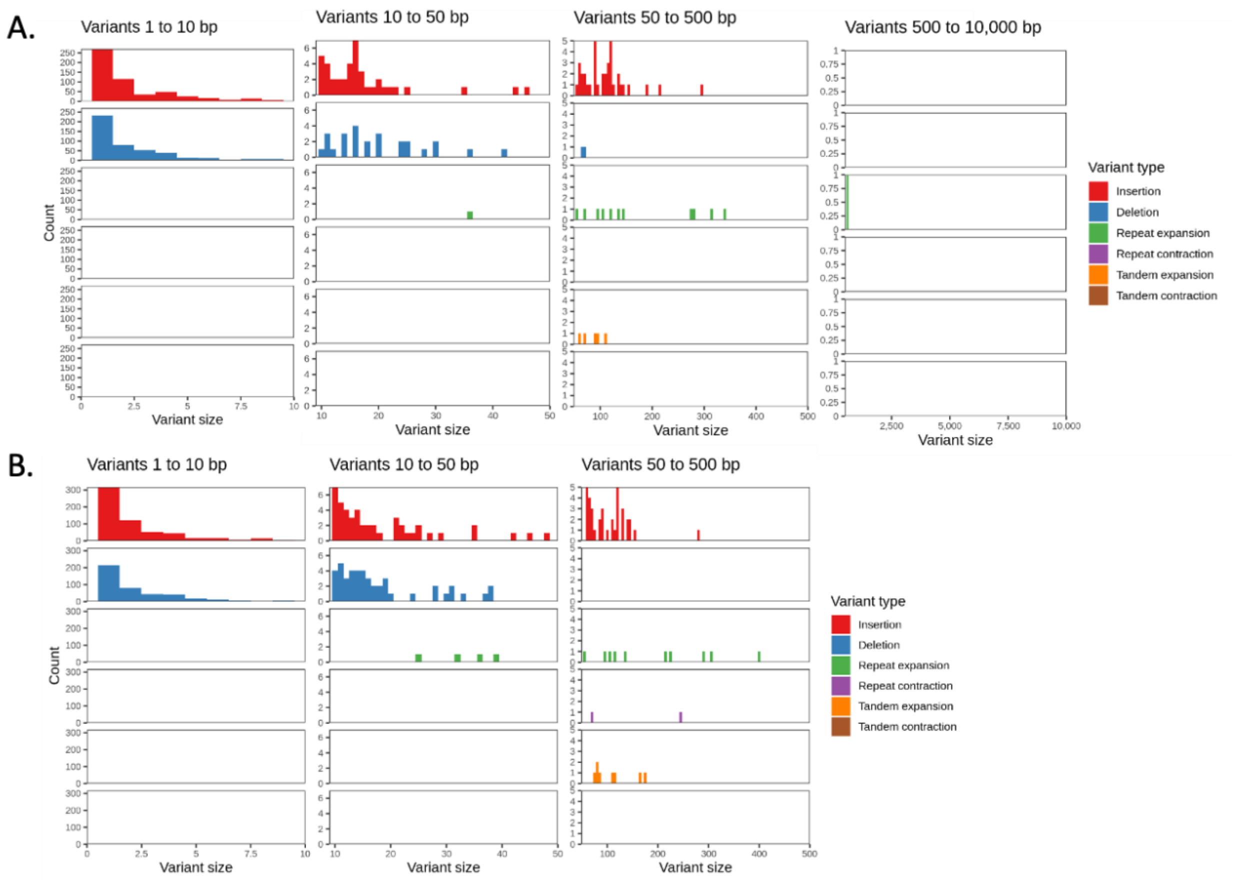

Fig S5. *Assemblytics* analysis of false SV composition for the MHConstructor *de novo* assembly of NA19240, as compared to phased, reference MHC haplotypes for NA19240. A) NA19240 MHC haplotype A, B) NA19240 MHC haplotype B. Categories of assembly SV errors described in legend.

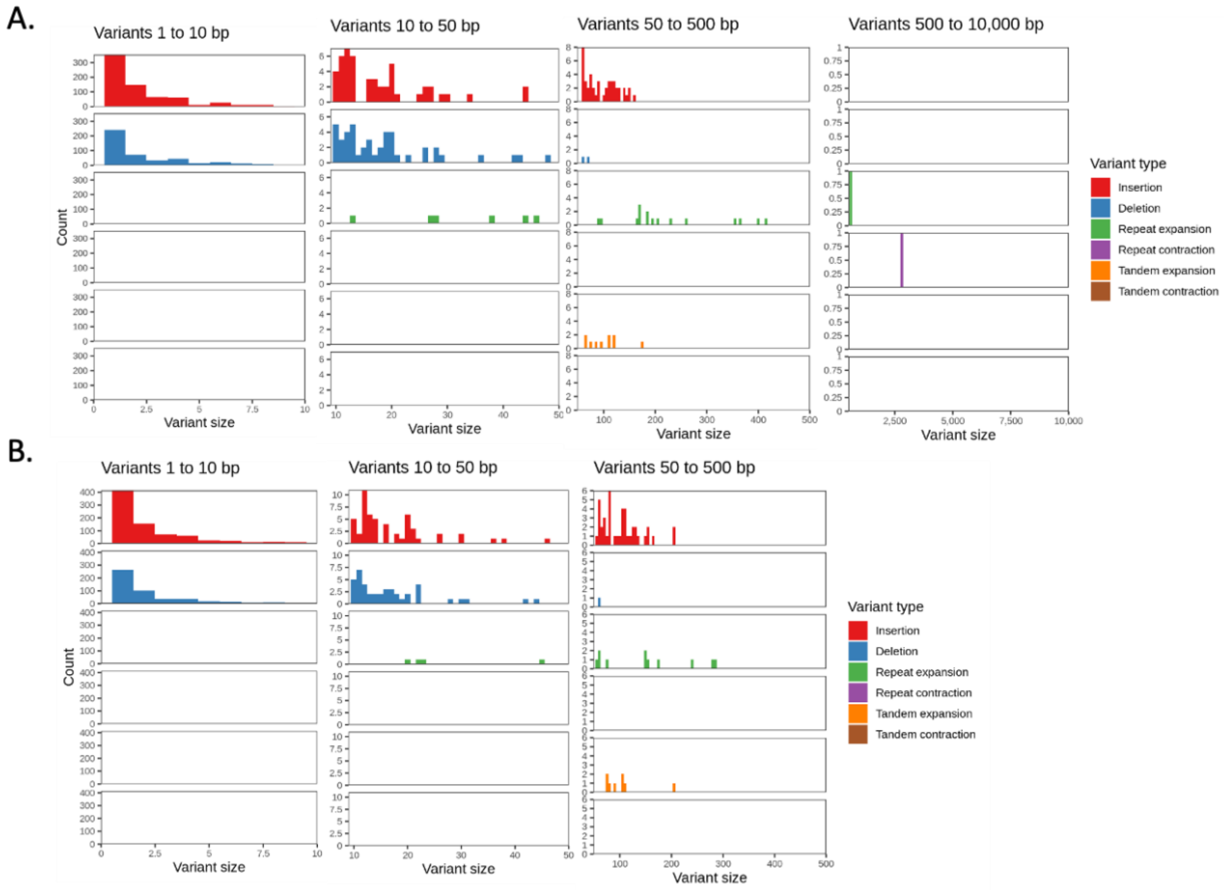

Fig S6. *Assemblytics* analysis of false SV composition for the MHConstructor *de novo* assembly of HG00621, as compared to phased, reference MHC haplotypes for NA20129. A) NA20129 MHC haplotype A, B) NA20129 MHC haplotype B. Categories of assembly SV errors described in legend.

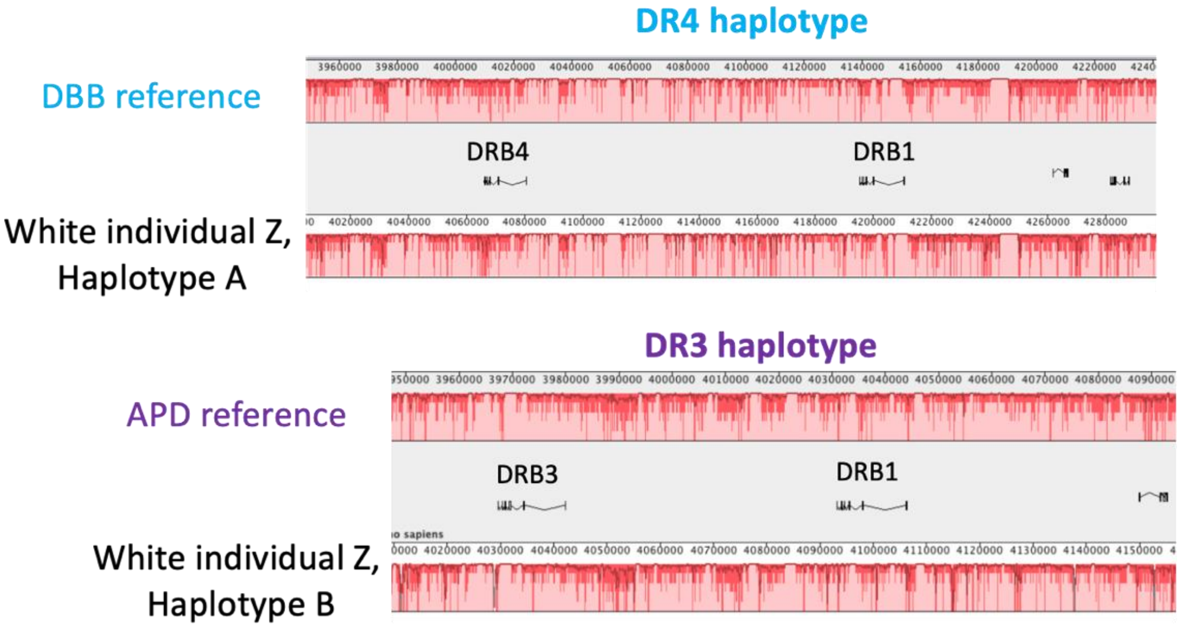

Fig S7. Mauve alignment plots confirm that the two DR haplotypes for heterozygous Individual Z are correctly assembled. In the top plot, the Individual Z sequence corresponding to the DR4 haplotype is displayed aligned against the DBB (DR4) published reference sequence (Houwaart *et al.*, 2022). In the bottom plot, the Individual Z sequence corresponding to the DR3 haplotype is displayed aligned against the APD (DR3) published reference sequence (Houwaart *et al.*, 2022). Light red indicates perfect alignment, dark red indicates presence of substitutions. HLA-DRB genes are labelled above their annotated coding regions.

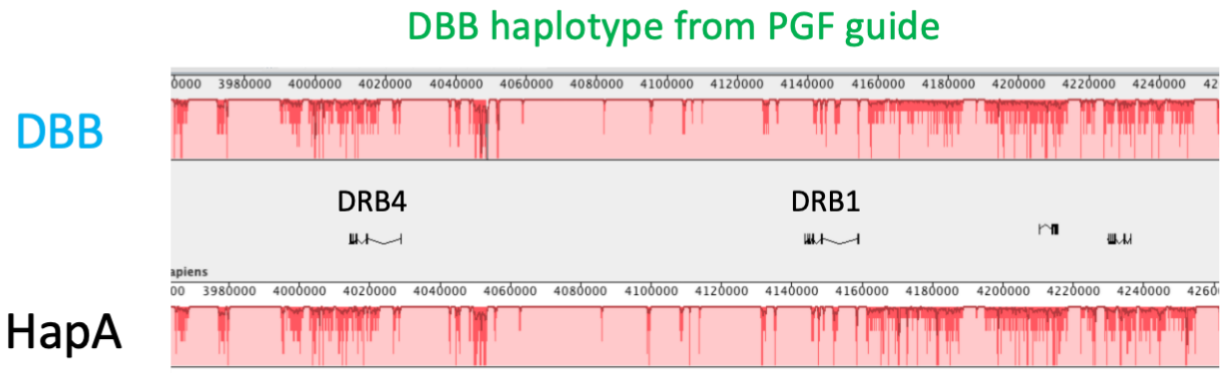

Fig S8. HLA Class II structure can be correctly assembled even when an incorrectly-match haplotype is used as the assembly guide, so long as the correct haplotype is used for scaffolding orientation.

MHConductor NA19240.1

GC level: 44.77 %
bases masked: 2744988 bp (53.95 %)

	number of elements*	length occupied	percentage of sequence
SINEs:	3396	821766 bp	16.15 %
ALUs	2798	728111 bp	14.31 %
MIRs	592	92718 bp	1.82 %
LINEs:	1731	1050336 bp	20.64 %
LINE1	1134	848383 bp	16.67 %
LINE2	524	181556 bp	3.57 %
L3/CR1	51	12950 bp	0.25 %
LTR elements:	992	617160 bp	12.13 %
ERVL	228	171551 bp	3.37 %
ERVL-MaLRs	358	152149 bp	2.99 %
ERV_classI	295	215025 bp	4.23 %
ERV_classII	47	56590 bp	1.11 %
DNA elements:	700	174889 bp	3.44 %
hAT-Charlie	396	93293 bp	1.83 %
TcMar-Tigger	151	54356 bp	1.07 %
Unclassified:	21	13236 bp	0.26 %
Total interspersed repeats:		2677387 bp	52.62 %
Small RNA:	87	7042 bp	0.14 %
Satellites:	4	3266 bp	0.06 %
Simple repeats:	991	45653 bp	0.90 %
Low complexity:	191	11161 bp	0.22 %

Base5 NA19240.1

GC level: 44.90 %
bases masked: 2895112 bp (57.11 %)

	number of elements*	length occupied	percentage of sequence
SINEs:	3446	849271 bp	16.75 %
ALUs	2828	751675 bp	14.83 %
MIRs	611	96462 bp	1.90 %
LINEs:	1761	1122194 bp	22.14 %
LINE1	1138	906985 bp	17.89 %
LINE2	545	193494 bp	3.82 %
L3/CR1	57	14535 bp	0.29 %
LTR elements:	1019	649642 bp	12.81 %
ERVL	232	177122 bp	3.49 %
ERVL-MaLRs	370	160049 bp	3.16 %
ERV_classI	299	228338 bp	4.50 %
ERV_classII	48	61061 bp	1.20 %
DNA elements:	728	182701 bp	3.60 %
hAT-Charlie	413	97084 bp	1.91 %
TcMar-Tigger	149	56228 bp	1.11 %
Unclassified:	20	21501 bp	0.42 %
Total interspersed repeats:		2825309 bp	55.73 %
Small RNA:	90	7288 bp	0.14 %
Satellites:	7	3455 bp	0.07 %
Simple repeats:	1013	46435 bp	0.92 %
Low complexity:	202	12146 bp	0.24 %

Fig S9. RepeatMasker annotation of repetitive sequence content in an MHConstructor WGS assembly and its corresponding phased MHC haplotype.

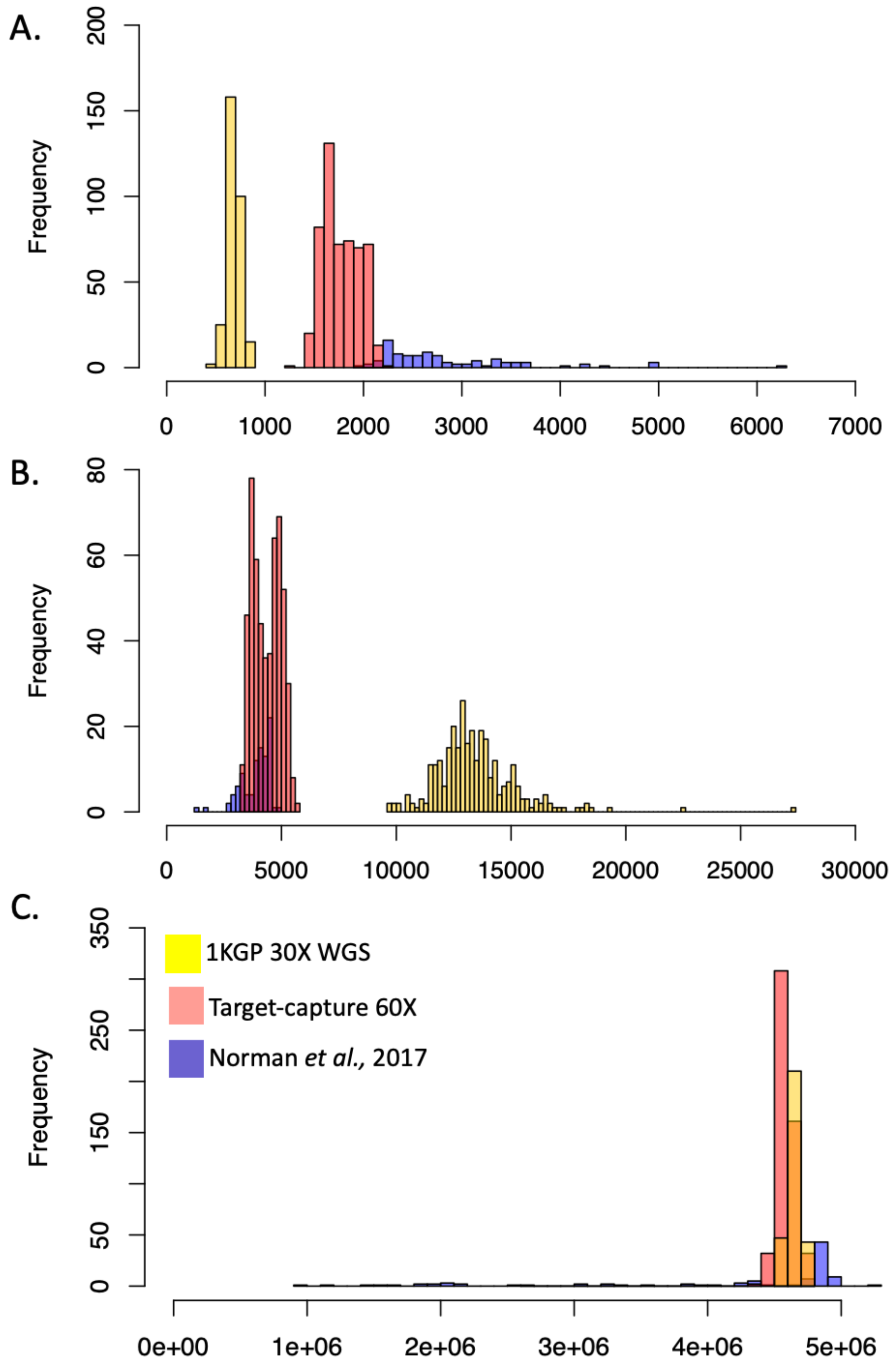

Fig S10. MHConstructor assembly metrics for target capture and WGS assemblies. Purple distributions represent published results from Norman *et al.*, 2017, n=95 haplotypes. Pink distributions represent assemblies generated from target-capture, 60x short read data, n=536 haplotypes. Yellow distributions represent assemblies generated from 1000Genomes WGS 30x short read data, n=300 haplotypes. A) Histogram of the number of contigs per assembly. B) Histogram of assembly N50 (bp), per assembly. C) Histogram of assembly lengths (bp).

.

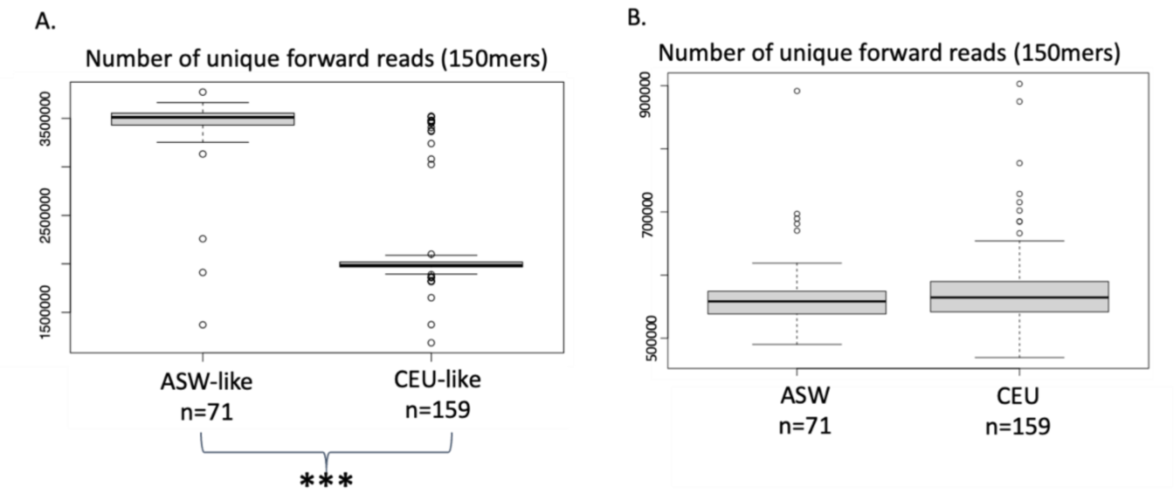

Fig S11. Nucleotide diversity within sequencing reads. A) Number of unique forward reads in subsampled ASW-like and CEU-like individuals, from target-capture approach. B) Number of unique forward reads in ASW and CEU populations, from WGS approach.
