# Supplementary material for: MHConstructor: a high-throughput, haplotype-informed solution to the MHC assembly challenge
Source: Genome Biol. 2024 Oct 17;25:274. doi: 10.1186/s13059-024-03412-6 (PMC11484429; doi:10.1186/s13059-024-03412-6)
Supplement: Supplementary file 6 — Additional file 6. Review history. [file 13059_2024_3412_MOESM6_ESM.docx]

**1^st^ round**

**Reviewer 1**

Dear authors,
I hereby supply my comments on your manuscript "MHConstructor: A high-throughput, haplotype-informed solution to the MHC assembly challenge". I congratulate you on a solid and exciting piece of work, which presents a valuable contribution to contemporary methods for MHC genotyping in humans. In particular, I like the concept of replacing trio-binning with phasing by comparing reads to a set of guide haplotypes. However, I also find this approach slightly risky, and I hope you can address some of my concerns that I list in my comments below.

I find the study relevant to Genome Biology readers, and I am convinced it has the potential to be published there after revision. However, I find that the manuscript is not ready in its current state. I present my comments below.

The best of luck!

Kind regards,
Jacob Roved
Department of Biology
Lund University

General comments

1. I find the concept of mapping de novo reads against guide MHC haplotypes very promising. However, there is an obvious challenge associated with this method, which is that you risk being limited by the availability of high-quality data on guide haplotypes that represent also the rarest variants in populations. The MHC region is dynamic and novel variation arises by mutations, recombination, micro-recombination, gene-conversion, etc. I therefore find it risky to assume that all MHC haplotypes can be safely mapped to the guide haplotypes that are incorporated in MHConstructor. I would like you to address this in the manuscript. How useful is MHConstructor for detecting novel variants, and what is the risk of missing such variants (or even worse, misidentifying them as similar to existing guide haplotypes) if they occur in de novo data sets?
2. In addition, I find it difficult to understand how MHConstructor phases nucleotide variation in individuals inferred to be homozygous, see my comment to L530-531 below. This should be clarified.
3. In continuation of my first comment, I would like you to also briefly discuss the potential consequences of limiting important large-scale studies of disease cohorts by mapping to known haplotype variants. You touch on it in L321-331 in the discussion, but I think you can invest a little more effort on this. It will improve the confidence in your method.
4. As my final general comment, I would like a bit more development of how reads that did not map to the guide haplotypes were evaluated. It is mentioned in L558-564 that such reads are de novo assembled separately and aligned to the rest of the human genome and for heterozygous individuals to the alt. MHC haplotype. Contigs that map outside the MHC or to the alt. haplotype are removed and remaining contigs combined with the reads that mapped. So, the unmapped reads stay in the data set (unless they are non-MHC), which is comforting. But this ties back to my first point above. How would MHConstructor deal with novel variants that don't map well to the guide haplotypes? Is there a risk that novel variants will produce poor data and may be excluded from downstream analyses, if they don't map well to the guide haplotypes? I think it would be relevant to clarify the fate of unmapped reads a bit more and include them in the discussion. I find MHConstructor a useful and powerful tool, but it is important to clarify whether its usage comes with a risk of limiting studies to MHC variation that conforms to known guide haplotypes.

Specific comments

L58-60 A few references would be appropriate.
L84 "resource prohibitive"?
L117-125 The language and coherence of this section can be improved.
L129 "We build from current short-read de novo assembly progress..." I suggest to improve the language.
150 "kmerGenie" - this is spelled in different ways throughout the manuscript. Please find the correct version and use it consistently.
L155-159 "For reads which mapped to the guide haplotype, best kmer size varied significantly across samples (stdev 4.15-21.19). Additionally, the number of reads evaluated impacts the best k-mer size identified, with greater sequencing depth allowing for larger kmer sizes. However, variability in predicted best kmer size between samples also increases with greater sequencing depth." Can you explain or hypothesize why you observed these patterns, especially those mentioned in the first and last of the three sentences? I find it especially intriguing that variability in predicted best kmer sizes should increase with greater sequencing depth.
L174-175 "we do find … does reach …" - do and does in the same sentence. I suggest to improve.
197 Please specify the abbreviation "SV". Also in the legend of Table 2.
206 "1000GenomesProject" - this is spelled in different ways throughout the manuscript. Please find the correct version and use it consistently.
L250 APD and DBB - please specify the abbreviations
L257-266 To which extent does the method assemble repetitive content by fitting short-read data to the reference haplotypes and to which extent is the inferred repeat data inherent in the analyzed de novo sequences? Is the method associated with a risk of artificially recreating the repetitive content of the reference haplotypes? Would a sample with deviant repetitive regions be inferred correctly and confidently? Or at least flagged as deviant?
L300-301 "This difference appears to also be reflected in the pre-assembly short read data, as compared between WGS and target-capture reads." I'm not sure that I follow this. Does it refer to the following sentences about the ratio of 150mers? If so, perhaps merge the sentences?
L318-344 I find this a bit messy. You mention three key areas, but then list four. Perhaps go for four, and insert a break at L342?
L359 "There are many additional parameters involved in this analysis which may require fine-tuning…" Could you provide a list of these in a table? And perhaps suggest how user should proceed with the fine-tuning? Is there any optimization involved? In that case, you may suggest how to carry it out. Do parameters interact? This information would be valuable to users of MHConstructor.
L376-377 You use "also" twice in the same sentence.
L377 "typical of most WGS is performed at" - check language
L383-384 "there are certain inherent technical limitations usually described in short-read assemblies" - what do you mean? I suggest to be more specific.
L386-387 "de novo short read assembly is not capable of discerning between C4A and C4B and their duplicates" - I'm curious why this is? Could you explain that a bit more? You go a long way to solve that problem (implementing C4Investigator), so it would be reasonable to dive into it a bit.
L392-393 These error rates are meaningful only in the context of providing a full picture of how the repetitive content is assembled. This refers back to my comment to L257-266. Are the repetitive regions inferred completely from the data or do the reference haplotypes influence the assembly?
L397-398 "Building on our preliminary SV predictions provided using output from MHConstructor, it is likely that future work may further refine these predictions through additional analysis using such algorithms." - this needs to be much more specific. I cannot follow.
L403 "Conclusions" - the following paragraphs don't read like a conclusion to me, so the header seems out of place. I miss an actual conclusions paragraph at the very end.
L403 "From these haplotype-specific assemblies…" - what does "these" refer back to?
L403 You start using the concept "variant information" out of the blue. It would improve the section if you would introduce this concept and its relevance to downstream data analysis with one or two sentences.
L427-431 I believe you already said something similar in the previous paragraph?
L432 "These high-resolution sequences…" - again beginning a paragraph with "these" that doesn't point to something.
L434 "causal variants" - causal of what?
L435 "assist in overcoming this challenge" - which challenge?
L436 "This tool…" - which tool?
L441 "This method…" - which method?
L441-443 I think you are overselling here. I cannot think of any non-human species whose MHC can be assumed to be mappable to a few select human MHC haplotypes. If you wish to make this claim, you need to thoroughly justify it with referenced background information.
L449 DNA from what tissue?
L461-463 Please specify which protocol you were following or provide a lot more detailed information.
L463 "0.8x ratio" - what was the DNA concentration of the solution that this ratio of beads is applied to?
L465 Which brand/type of magnetic beads?
L465 + 471 Please specify how the amplification was done. Primers? PCR settings?
L466-467 "via ultrasonic acoustic energy" - please specify machine and settings.
L468-470 How were the probes designed? Or were they part of a kit? Please specify or provide a reference.
L470 "streptavidin magnetic beads" - brand/type and amount?
L471 How was purification done?
L489-490 "Reads which did not map to these MHC regions were extracted separately and used in later stages of analysis." - This needs to be clarified. Do you mean the rest of the genomes?
L500-504 "Since HLA-DRB1 is in known to be in strong linkage disequilibrium with HLA-DRB3, -DRB4 and -DRB5 (Rollini et al. 1985; Maiers et al. 2007), we infer that the first field genotypes of HLA-DRB3, -DRB4 and -DRB5 will correspond to previously established HLA-DRB1 haplotypes (Houwaart et al. 2023). In this way, we determine the HLA class II haplotype(s) for each individual." - is this a safe assumption? It is central to the method because the reads are subsequently mapped to the inferred haplotype. I think the background should be described in more detail and that it could be relevant to justify this assumption with some kind of quantification or assessment.
L515-516 "minor code alterations…" - I suggest to specify these in supplementary materials.
L530-531 "if an individual is homozygous at HLA-DRB1" - at which resolution? Amino acid? In L497 you mention first field resolution, which in my understanding is amino acid level. Individuals can be homozygous at the amino acid level, but heterozygous at the nucleotide level. How does MHConstructor deal with phasing nucleotide variation in individuals inferred to be homozygous by MHConstructor?
L588-589 "randomly subsampling pairs of reads at 200k, 500k, 1M, 2M, and 3M intervals" - I cannot follow. What is meant by e.g. a 200k interval?
L603 "data from individuals from the30xthat had their MHC phased sequence assembled with - technology" - what is this sentence?
L617-618 "the consensus sequence(s) from each assembly" - what are these?
L618-619 "Following characterization, repeat regions were then masked" - what does this mean?

**Reviewer 2**

In the manuscript titled "MHConstructor: A high-throughput, haplotype-informed solution to the MHC assembly challenge ", the authors present a reference-guided assembly pipeline for constructing the major histocompatibility complex (MHC) from short reads. The pipeline integrates multiple tools, and some modifications have been made to enhance the performance. The MHC region is complex, and while third-generation long reads are preferable for MHC assembly, short reads offer cost advantages. Reference genomes are commonly used to assist in assembling short reads. The algorithm's core contribution lies in using multiple MHC reference sequences to guide the assembly process. However, the manuscript lacks sufficient evidence to support the proposed improvement. First, I recommend comparing MHConstructor with other reference-guided methods, such as Lischer and Shimizu (2017), to demonstrate the benefits of using multiple reference genomes. Second, the evaluation method in sections 3.1 and 3.2, which relies on datasets associated with the reference sequences, may overestimate the tool's performance.

Other issues:
(1) Line 603-604, There are some spelling errors..
(2) Line 117 "Recent work" and Line 191 "base on our previous finding" need references.
(3) Line 552, The item "superblock" lacks a description.
(4) Supplemental Table S5 contains duplicate content.

**Authors’ response**

Reviewer #1

1. I find the concept of mapping de novo reads against guide MHC haplotypes very promising. However, there is an obvious challenge associated with this method, which is that you risk being limited by the availability of high-quality data on guide haplotypes that represent also the rarest variants in populations. The MHC region is dynamic and novel variation arises by mutations, recombination, micro-recombination, gene- conversion, etc. I therefore find it risky to assume that all MHC haplotypes can be safely mapped to the guide haplotypes that are incorporated in MHConstructor. I would like you to address this in the manuscript. How useful is MHConstructor for detecting novel variants, and what is the risk of missing such variants (or even worse, misidentifying them as similar to existing guide haplotypes) if they occur in de novo data sets?

We thank the reviewer for these comments, which provide an opportunity to improve clarity in the manuscript. We would like to clarify our use of ‘de novo’ and ‘mapping’ here. We use it in the same context as Lischer and Shimizu, 2017, the original paper whose source code and assembly algorithm are implemented in MHConstructor. Their work was designed specifically for the challenge of assembling the genome of a species that does not yet have a reference genome. They reason that the reference genome of a closely related species, while containing too much variation to meaningfully align reads to, still shares enough sequence homology with its sister species to help inform, or guide, the de novo assembly process of the novel genome. We chose the algorithm for a similar reason, since it would be sensitive to novel variation, while using the few quality MHC reference sequences that are available to inform and simplify the de novo assembly process.

‘de novo’ sequence assembly is a commonly used method of reference-free sequence assembly (Miller et al. 2010; Nagarajan and Pop 2013; Koren et al. 2018; Dida and Yi 2021; Zhang et al. 2021). It functions by breaking sequencing reads into smaller sequence fragments of length k (kmers) and then connecting kmers into graph structures based on sequence overlap (Miller et al. 2010; Nagarajan and Pop 2013). These structures are known as DeBrujin graphs and represent the most likely path through overlapping sequence fragments (Miller et al. 2010; Nagarajan and Pop 2013). These graphs are then condensed into longer assembled sequence fragments known as contigs. The primary benefit of this approach is that it does not rely on alignment to a reference sequence and therefore is much more likely to capture previously undescribed variation (Miller et al. 2010; Nagarajan and Pop 2013) Further, de novo assembly has already been successfully used to generate MHC sequences, examples of which have been described in the Introduction. However, none of these papers have published a reproducible pipeline or software package for the assembly process. This is one of the key motivations for creating MHConstructor. By developing a high throughput and reproducible tool for de novo MHC sequence assembly, we hope that others will be able to generate their own de novo MHC sequences as well.

To clarify the use of ‘mapping’, we only map (align) reads to the major MHC haplotypes as a means to bin (or separate) reads by haplotype. Haplotype-binning has already been successfully used in several MHC assembly contexts (Koren et al. 2018; Wang et al. 2023). We chose the set of guide MHC haplotypes that capture the primary categories of structural variation observed in the HLA class II region. As seen in Jensen et al., 2017 and Houwaart et al., 2023, this region, as well as C4 (which we account for by including C4Investigator), are the primary sources of structural variation in the MHC that have been observed thus far. Because of this, we infer that the use of these major, known categories of SVs are represented by the six major haplotypes characterized by Houwaart et al., 2023 and thus, are sufficient to assist in structural assembly of the HLA class II region. The structural variation in this region would likely thwart the de novo assembly process if assembled collectively. Therefore, we circumvent this issue by binning reads from haplotypes associated with differing structural variation prior to de novo assembly. Reads which were alignable to each haplotype are then extracted from those alignments and de novo assembled separately for each haplotype, as described in the previous paragraph. As mentioned before, this method is already published in Lischer and Shimizu, 2017. The guide MHC haplotype sequences are then used at a second point in the MHConstructor assembly process, which is known as scaffolding. During this stage, the de novo assembled contigs are ordered and oriented with respect to a reference sequence, in this case, the guide MHC haplotypes. This is done to orient the de novo assembled contigs, not to call variation. Scaffolding is a well-established practice and the software we use to do this, RagTag, is commonly used.

Reads which did not align to a guide haplotype can be assumed to represent novel variation of a large structural nature (>150bp) and are captured through a separate de novo assembly process, only using reads which do not map to the guide haplotype. However, because of the near impossibility of meaningfully including these contigs into the final, scaffolded sequence assembly in a high throughput way, we have left these sequences out and encourage users to investigate these sequences in a post-hoc analysis that will likely require manual curation and evaluation.

We have revised the introduction and added additional clarification of these definitions to the text to make our use of haplotype binning, de novo assembly and scaffolding more easily understood. We have also added further explanation of why such an approach was chosen and the benefits it offers for accessing novel MHC variation. To demonstrate the viability of this approach for identifying novel variation not observed in the guide haplotype, we have added two additional Supplemental Tables, S9 and S10, with corresponding manuscript text added to Results 3.3 and lines 666- 677 of the Methods.

2. In addition, I find it difficult to understand how MHConstructor phases nucleotide variation in individuals inferred to be homozygous, see my comment to L530-531 below. This should be clarified.

Thank you for this opportunity to clarify. These guide haplotypes are not used for the purposes of formal phasing. As mentioned in our response to comment #1, we use the guide haplotype sequences in the way described by Lischer and Shimizu, 2017. Our primary goal in assigning each individual either heterozygous or homozygous guide MHC haplotypes, is to separate reads associated with the major categories of structural variation at the HLA class II region, for subsequent de novo assembly. The primary manifestations of this HLA class II variation are represented within these six major haplotype sequences (Houwaart et al., 2023). Furthermore, for the purposes of disease association studies, phased variation is not required.

3. In continuation of my first comment, I would like you to also briefly discuss the potential consequences of limiting important large-scale studies of disease cohorts by mapping to known haplotype variants. You touch on it in L321-331 in the discussion, but I think you can invest a little more effort on this. It will improve the confidence in your method.

Thank you for pointing out this opportunity to improve confidence in MHConstructor. We would like to re-emphasize that we are not calling variation from read alignment to known haplotype variants. We only use read mapping to major haplotypes as a means to bin reads by HLA class II structural haplotype. Those reads are then extracted from those alignments and de novo assembled. Thus, our sequences are generated from de novo assembly and therefore we are not limiting ourselves to just the variation that is represented in the guide haplotypes.

We do, however, acknowledge the impact of variant size on our ability to detect novel variation, due to limitations inherent in the sequencing method. The length of paired end short read sequence data that we have developed this approach for is 150 bp. Therefore, even with a de novo assembly method, any novel variation longer than 150bp has inherently lower confidence, especially if it is part of a repetitive sequence element. This is a well known limitation of working with short read data and is true of large novel variation in any short read dataset. We appreciate your encouragement to more clearly articulate the limitations of the approach, and have added more detail to the Discussion, lines 425-440 which better communicates this.

4. As my final general comment, I would like a bit more development of how reads that did not map to the guide haplotypes were evaluated. It is mentioned in L558-564 that such reads are de novo assembled separately and aligned to the rest of the human genome and for heterozygous individuals to the alt. MHC haplotype. Contigs that map outside the MHC or to the alt. haplotype are removed and remaining contigs combined with the reads that mapped. So, the unmapped reads stay in the data set (unless they are non-MHC), which is comforting. But this ties back to my first point above. How would MHConstructor deal with novel variants that don't map well to the guide haplotypes? Is there a risk that novel variants will produce poor data and may be excluded from downstream analyses, if they don't map well to the guide haplotypes? I think it would be relevant to clarify the fate of unmapped reads a bit more and include them in the discussion. I find MHConstructor a useful and powerful tool, but it is important to clarify whether its usage comes with a risk of limiting studies to MHC variation that conforms to known guide haplotypes.

Thank you for the opportunity to clarify the assembly method implemented in MHConstructor. As described above, the guide haplotypes are used to bin (separate) the reads so that reads which are unique to a given HLA class II region can be extracted from alignment and de novo assembled separately. Any reads which are not able to be aligned to a given guide haplotype, are then de novo assembled separately, into contigs of putatively novel content. This is done to capture novel structural variation that is larger than the read length and not observed in the guide haplotype. Contigs generated from reads that didn’t map to a guide haplotype are scanned for off- target mapping and removed if located elsewhere in the human genome or on the alternative guide haplotype. Contigs are then merged with the contigs assembled from reads that did map well to the guide haplotype for the rest of the pipeline, as described in Lischer and Shimizu et al., 2017. Given that those contigs likely contain putative novel sequence content, they are also unlikely to be scaffolded (placed/aligned) successfully against the guide haplotype. In this case, contigs which RagTag cannot scaffold are output as separate fasta sequence entries from the continuous, scaffolded assembly sequence. There is currently no way to automatically infer where in the assembly these novel contigs should be placed in a high throughput manner. Therefore, if users are interested in including the novel contigs, we recommend manual, post-hoc evaluation and placement of these sequences.

To clarify this in the manuscript, we have added the following statement to the Methods section, lines 622-627: “Any de novo assembled contigs that RagTag is not able to place on the BMH guide sequence are output as separate fasta records in the same file as the continuous, consensus sequence. There is currently no way to automatically infer where in the assembly these novel contigs should be placed in a high throughput manner. Therefore, if users are interested in including the novel contigs, manual, post- hoc evaluation and placement of these sequences is required.”

Specific comments:

L58-60 A few references would be appropriate.

References have been added.

L84 "resource prohibitive"?

We have re-phrased the statement to be clearer: “computational complexity and cost of long read sequencing at population scale”

L117-125 The language and coherence of this section can be improved.

This section has been revised.

L129 "We build from current short-read de novo assembly progress..." I suggest to improve the language.

We have revised the language to say: “While many previous advances have been made in handling the problem of MHC sequence assembly…”.

150 "kmerGenie" - this is spelled in different ways throughout the manuscript. Please find the correct version and use it consistently.

Thank you for pointing this out, we have replaced all instances with the correct name “KmerGenie”.

L155-159 "For reads which mapped to the guide haplotype, best kmer size varied significantly across samples (stdev 4.15-21.19). Additionally, the number of reads evaluated impacts the best k-mer size identified, with greater sequencing depth allowing for larger kmer sizes. However, variability in predicted best kmer size between samples also increases with greater sequencing depth." Can you explain or hypothesize why you observed these patterns, especially those mentioned in the first and last of the three sentences? I find it especially intriguing that variability in predicted best kmer sizes should increase with greater sequencing depth.

Thank you for pointing this out. We suspect that this may be because the inclusion of a greater read depth increases the probability of including reads representing variants that may be unique to each individual. Therefore, the more reads, the more variability in best predicted kmer size, as each individual may have unique patterns of variation that lower coverage sampling may not pick up. We have added this interpretation to section 2.1 of the Results text.

L174-175 "we do find … does reach …" - do and does in the same sentence. I suggest to improve.

This has been revised.

197 Please specify the abbreviation "SV". Also in the legend of Table 2.

Thank you for correcting this. We have added an in-text correction. Table 2 already contains the full definition of SV in the legend underneath the table.

206 "1000GenomesProject" - this is spelled in different ways throughout the manuscript. Please find the correct version and use it consistently.

Thank you for identifying this, it has been corrected to read “1000 Genomes Project” at every instance.

L250 APD and DBB - please specify the abbreviations

We thank the reviewer for their concern with making our manuscript as accessible as possible. However, ‘APD’ and ‘DBB’ are the names of the cell lines from which the DNA was obtained. They are canonically referenced in this way (Horton et al. 2008).

L257-266 To which extent does the method assemble repetitive content by fitting short-read data to the reference haplotypes and to which extent is the inferred repeat data inherent in the analyzed de novo sequences? Is the method associated with a risk of artificially recreating the repetitive content of the reference haplotypes? Would a sample with deviant repetitive regions be inferred correctly and confidently? Or at least flagged as deviant?

As previously described, these assemblies are not generated from alignment to the guide haplotype. All sequences, including repetitive sequences, are inferred de novo, ie; reference-free, overlapping short read k-mers using a De Brujin Graph. So, while there is no risk of overly inferring the repeat structure of the guide sequence, there is a risk of improperly inferring the size of a repetitive element, when the length of the repeat is longer than the read fragment. This is a commonly understood limitation of using de novo assembly algorithms with short read sequencing data. Knowing this as an inherent limitation of the method, we sought to describe this error rate in several ways. We refer the Reviewer to Tables 2 and 3, Supplemental Figures S4-S6 and Supplemental Table S9 for quantification of MHConstructor error rate at repetitive regions.

If users would prefer to remove any chance of repeat-associated error from their assemblies, they can perform repeat masking, where a tool, such a RepeatMasker is used to first identify, and then mask repeat sequences by replacing them with ‘N’, so that they are not read as sequence content by downstream analysis tools. This is common practice in sequence assembly and we describe this process in the final section of the Methods. The section now reads: “To characterize the repetitive elements located within novel MHC assemblies, we applied repeatMasker v4 (http://www.repeatmasker.org) to the consensus sequence(s) from each assembly, using default parameters. Following characterization, repeat regions were then masked. Masking is the process of replacing the nucleotide sequence of regions identified to be repetitive elements with ‘N’, in place of each nucleotide base. This was also carried out with repeatMasker using default settings.”

L300-301 "This difference appears to also be reflected in the pre-assembly short read data, as compared between WGS and target-capture reads." I'm not sure that I follow this. Does it refer to the following sentences about the ratio of 150mers? If so, perhaps merge the sentences?

Thank you for directing us to this confusing paragraph. It has been re-written as such: “There was a corresponding difference in terms of kmer diversity between these two populations as well. In the target-capture short read fragments, the mean number of unique 150mers per ASW-like reads was significantly higher than the mean unique 150mers in the CEU-like reads (p-value < 2.2e-16). Interestingly, when comparing these same assembly metric distributions between the CEU and ASW assemblies generated from 30X 1000 Genomes Project WGS data, no differences were observed in contig number (p-value = 0.3184) or N50 length (p-value= 0.2629), though total assembly length was slightly higher in the CEU population (p-value= 0.004869). Additionally, there was no observable difference in number of unique WGS short read 150mers between the two populations (p=0.5212) (Supplemental Figure S10). To ensure that the increased kmer diversity difference between populations observed in the target-capture data were not an artifact of the larger sample size (n=536 haplotypes), relative to the WGS 1000 Genomes Project samples (n=300 haplotypes), we performed random subsampling of the target-capture individuals to achieve equivalent sample sizes. Even at a smaller sample size (n=300), there were still significantly more unique 150mers in the target-capture short reads for ASW-like individuals (p-value < 2.2e-16).”

L318-344 I find this a bit messy. You mention three key areas, but then list four. Perhaps go for four, and insert a break at L342?

Thank you for finding this opportunity to increase clarity. We have chosen to re-orient the points with respect to the revised structure of the introduction. In this paragraph, 358-363, we have replaced “reproducibility and accessibility” with a single word, usability.

L359 "There are many additional parameters involved in this analysis which may require fine-tuning…" Could you provide a list of these in a table? And perhaps suggest how user should proceed with the fine-tuning? Is there any optimization involved? In that case, you may suggest how to carry it out. Do parameters interact? This information would be valuable to users of MHConstructor.

Thank you for this opportunity to increase useability of MHConstructor. We have added a statement to the manuscript that refers users to the control.txt file associated with the MHConstructor Github release, in lines 639-643. It reads: “Assembly parameters for multithreading, insert size, expected coverage, de novo assembly kmer length and starting number of reads are listed in the text editable control.txt file included in the MHConstructor software package. This file is built into the Singularity image and must be edited by the user to be appropriate for each individual analysis.”

We also note that in Results section 2 we describe our parameter optimization process regarding these parameters. Users can reference and replicate this section when deciding how to evaluate their own assemblies. As far as parameter interactions and their impact on assembly quality, we refer the Reviewer to Table 1, Supplemental Tables S1-S4 and Supplemental Figures S1- S2.

L376-377 You use "also" twice in the same sentence.

This has been corrected.

L377 "typical of most WGS is performed at" - check language

This has been revised.

L383-384 "there are certain inherent technical limitations usually described in short- read assemblies" - what do you mean? I suggest to be more specific.

This has been revised to say: “An inherent limitation of this method comes from the nature of short read sequence data. Since the sequence fragments used in short read sequencing are generally shorter than 200bps in length., this can result in assembly errors when the length of a repetitive motif is longer than the length of the read fragment (Alkan et al. 2011; Dida and Yi 2021). “

L386-387 "de novo short read assembly is not capable of discerning between C4A and C4B and their duplicates" - I'm curious why this is? Could you explain that a bit more? You go a long way to solve that problem (implementing C4Investigator), so it would be reasonable to dive into it a bit.

Since C4A and C4B are identical at the nucleotide level, with the exception of four codons, it is almost impossible for de novo assembly algorithms to meaningfully identify the correct path through elements with such a high sequence identity (Marin et al. 2024). Similarly, copy number variation is unlikely to be accurately captured from short read sequence assembly alone and requires an estimation of coverage depth. For these reasons, we have implemented the tool C4Investigator (Marin et al. 2024), which is designed to distinguish between the presence of C4A and C4B, long and short forms, and copy numbers. To clarify, a more complete description has been added to lines 431-436.

L392-393 These error rates are meaningful only in the context of providing a full picture of how the repetitive content is assembled. This refers back to my comment to L257-266. Are the repetitive regions inferred completely from the data or do the reference haplotypes influence the assembly?

Please see our response to the L257-266 comment.

L397-398 "Building on our preliminary SV predictions provided using output from MHConstructor, it is likely that future work may further refine these predictions through additional analysis using such algorithms." - this needs to be much more specific. I cannot follow.

This section has been removed, as we agree that it was a source of confusion.

L403 "Conclusions" - the following paragraphs don't read like a conclusion to me, so the header seems out of place. I miss an actual conclusions paragraph at the very end.

Thank you for pointing this out. This last paragraph is meant to be summary of possible future directions for this work. However, we acknowledge that this was not as clear in the original writing. Our Discussion section has now been revised for a more consistent flow in the following order: Conclusions from the manuscript, limitations and future directions.

L403 "From these haplotype-specific assemblies…" - what does "these" refer back to?

Thank you for pointing this out. This section has been re-structured as “Future applications” for increased clarity.

L403 You start using the concept "variant information" out of the blue. It would improve the section if you would introduce this concept and its relevance to downstream data analysis with one or two sentences.

Thank you for pointing this out. This section has been re-structured as “Future applications” for increased clarity. We introduce the concept of variant-calling here, pointing towards future work, as this was not performed in the context of this manuscript. This paragraph can be found in lines 833-936.

L427-431 I believe you already said something similar in the previous paragraph?

This has been removed from the previous paragraph.

L432 "These high-resolution sequences…" - again beginning a paragraph with "these" that doesn't point to something.

This has been revised to be more specific.

L434 "causal variants" - causal of what?

Thank you for pointing this out. This section has been removed as part of the paragraph restructuring. The revised future applications paragraph can be found in lines 425-448.

L435 "assist in overcoming this challenge" - which challenge?

Thank you for pointing this out. This section has been removed as part of the paragraph restructuring. The revised future applications paragraph can be found in lines 425-448.

L436 "This tool…" - which tool?

Thank you for pointing this out. This section has been removed as part of the paragraph restructuring. The revised future applications paragraph can be found in lines 425-448.

L441 "This method…" - which method?

Thank you for pointing this out. This section has been removed as part of the paragraph restructuring. The revised future applications paragraph can be found in lines 425-448.

L441-443 I think you are overselling here. I cannot think of any non-human species whose MHC can be assumed to be mappable to a few select human MHC haplotypes. If you wish to make this claim, you need to thoroughly justify it with referenced background information.

Thank you for this opportunity to clarify. It was not meant to be implied that human MHC guide sequences would be used. We have added additional text qualifying that this could be applicable to species which already have one or more fully assembled reference MHC sequences from their own species’ genome.

L449 DNA from what tissue?

Thank you for identifying this opportunity to clarify. The sentence now reads: “DNA from peripheral blood mononuclear cells (PBMCs) of healthy individuals (Oksenberg et al. 2004; Hollenbach et al. 2019) was obtained for target-capture sequencing.”

L461-463 Please specify which protocol you were following or provide a lot more detailed information.

The protocol is specified in Methods: Library Preparation Enzymatic Fragmentation Kit 2.0, Twist Bioscience.

L463 "0.8x ratio" - what was the DNA concentration of the solution that this ratio of beads is applied to?

Every sample started with 100 ng of DNA. The concentration at this step is irrelevant, not measured, and not required by the manufacturer’s protocol, as it is directly proportional to the initial DNA input amount. We have sequenced over 10,000 samples with many different kits, and the concentration at this step was never measured in any protocol, as the AMPure beads provide high recovery and can bind at least 7 μg of DNA per microliter of reagent.

L465 Which brand/type of magnetic beads?

As mentioned in the previous line, the beads were AMPure XP (Beckman Coulter). We have repeated the information now in this line.

L465 + 471 Please specify how the amplification was done. Primers? PCR settings?

The PCR instructions are stated in the Library Preparation Enzymatic Fragmentation Kit 2.0 protocol provided by the manufacturer. The name of the kit and vendor are stated in this section.

L466-467 "via ultrasonic acoustic energy" - please specify machine and settings. Instrument make and model were specified.

No specific settings are needed.

L468-470 How were the probes designed? Or were they part of a kit? Please specify or provide a reference.

We have clarified that we used a Twist Custom Panel by Twist Biosciences.

L470 "streptavidin magnetic beads" - brand/type and amount?

Streptavidin beads are included in the Twist Target Enrichment kit (Twist Bioscience), mentioned in this section. More details can be found in the manufacturer's protocol.

L471 How was purification done?

“AMPure beads” was repeated again in this line.

L489-490 "Reads which did not map to these MHC regions were extracted separately and used in later stages of analysis." - This needs to be clarified. Do you mean the rest of the genomes?

Thank you for this point of clarification. Every .bam (alignment) file for a given 1000 Genomes Projects individual contains the reads which did not map to the reference. These can be extracted using samtools view -u. The text has been updated.

L500-504 "Since HLA-DRB1 is in known to be in strong linkage disequilibrium with HLA-DRB3, -DRB4 and -DRB5 (Rollini et al. 1985; Maiers et al. 2007), we infer that the first field genotypes of HLA-DRB3, -DRB4 and -DRB5 will correspond to previously established HLA-DRB1 haplotypes (Houwaart et al. 2023). In this way, we determine the HLA class II haplotype(s) for each individual." - is this a safe assumption? It is central to the method because the reads are subsequently mapped to the inferred haplotype. I think the background should be described in more detail and that it could be relevant to justify this assumption with some kind of quantification or assessment.

As clarified in our responses to the primary concerns, assemblies are generated in a reference-free, de novo manner. Therefore, the guide sequence is representative enough to use for read binning prior to de novo assembly. Further, it is a safe assumption that there is strong linkage disequilibrium across the human MHC, as it has been amply described in decades of literature. Please see corresponding citations for additional explanations.

L515-516 "minor code alterations…" - I suggest to specify these in supplementary materials.

Thank you for identifying this opportunity for improved transparency. There are currently three statements already in the Methods section which describe modifications to the original algorithm. The first two are described in lines 588-598: “Both categories of reads are then extracted and de novo assembled into contigs via de Bruijn kmer graphs, as implemented by velvet (Zerbino and Birney 2008). Velvet was chosen instead of other assemblers described in the original publication (Lischer and Shimizu 2017) for its use of de Bruijn graphs and because its faster speed makes it better suited to high throughput applications in the context of large disease cohorts. The resulting contigs from the un-mapped sequence are aligned to the rest of the human reference genome (HG38) and, for heterozygous individuals, to the alternative MHC haplotype, using minimap2 (Li 2018). Any contigs that map outside of the MHC, or map to the alternative MHC haplotype reference are removed. This step is an addition to the original algorithm.” The third alteration is described in lines 603-607 and reads “Original reads are mapped onto the contigs and GATK HaplotypeCaller (McKenna et al. 2010; DePristo et al. 2011; Van der Auwera et al. 2013) is used to perform localized error-correction. This represents a deviation from the tool described in the original publication (Lischer and Shimizu 2017), as the GATK error-correction tool originally chosen is no longer supported by GATK.”

We have added one additional statement, describing the exclusion of a secondary round of de novo assembly for reads which did not map to the newly generated supercontigs 607-610: “In the interest of reducing assembly time for use in large cohorts, a secondary round of de novo assembly with the reads which did not map to merged supercontigs was excluded. This can be found in Step 5 of the original assembly pipeline (Lischer and Shimizu 2017).”

Further, we would like to add that all code for MHConstructor is publicly available on Github (https://github.com/Hollenbach-lab/MHConstructor) and can be compared directly to the original source code from Lischer and Shimizu, 2017.

L530-531 "if an individual is homozygous at HLA-DRB1" - at which resolution? Amino acid? In L497 you mention first field resolution, which in my understanding is amino acid level. Individuals can be homozygous at the amino acid level, but heterozygous at the nucleotide level. How does MHConstructor deal with phasing nucleotide variation in individuals inferred to be homozygous by MHConstructor?

Homozygous here refers to first field genotypes HLA-DRB1 and the text has been updated to specify this. As described previously, this level of genotype is sufficient to infer which category of major HLA class II structural variation will be present on the chromosome. As described above, we are not using guide sequences for phasing.

L588-589 "randomly subsampling pairs of reads at 200k, 500k, 1M, 2M, and 3M intervals" - I cannot follow. What is meant by e.g. a 200k interval?

Thank you for identifying this point of clarification. These intervals refer to numbers of subsampled read pairs (200,000 read pairs, 500,000 read pairs, etc.) used to evaluate the impact of coverage depth on various assembly metrics. The text has been updated and reads: “Following algorithm development, the effect of coverage depth onassembly time, computational cost, and assembly quality was evaluated by randomly subsampling pairs of reads to represent different coverage depths at 200,000 read pairs, 500,000 read pairs, 1 million read pairs, 2 million read pairs, and 3 million read
pairs, for each sample.”

L603 "data from individuals from the30xthat had their MHC phased sequence
assembled with - technology" - what is this sentence?
Thank you for catching this. This sentence was an artifact from the editing process and
has been removed.

L617-618 "the consensus sequence(s) from each assembly" - what are these?
Consensus sequences are the single continuous sequence that is produced following
assembly scaffolding and are a well-established means of representing sequence
assemblies in a single sequence, rather than as a collection of disconnected contigs
(Chu et al. 2018; Lang 2022).

L618-619 "Following characterization, repeat regions were then masked" - what does
this mean?
This means that a version of these assemblies was generated where the repeat-
associated error is removed from the assembly. This is done by first annotating the
repeat elements by comparison to a reference library of known repeat elements, and
then replacing the nucleotide bases represented by ‘A’,’T’,’C’ or ‘G’ at those positions
with ‘N’. This process is commonly known as ‘masking’ and all stages are performed by
RepeatMasker using default command settings. We have added this additional
explanation to this paragraph of the Methods.

Reviewer #2

In the manuscript titled "MHConstructor: A high-throughput, haplotype- informed solution to the MHC assembly challenge ", the authors present a reference- guided assembly pipeline for constructing the major histocompatibility complex (MHC) from short reads. The pipeline integrates multiple tools, and some modifications have been made to enhance the performance. The MHC region is complex, and while third- generation long reads are preferable for MHC assembly, short reads offer cost advantages. Reference genomes are commonly used to assist in assembling short reads. The algorithm's core contribution lies in using multiple MHC reference sequences to guide the assembly process. However, the manuscript lacks sufficient evidence to support the proposed improvement. First, I recommend comparing MHConstructor with other reference-guided methods, such as Lischer and Shimizu (2017), to demonstrate the benefits of using multiple reference genomes. Second, the evaluation method in sections 3.1 and 3.2, which relies on datasets associated with the reference sequences, may overestimate the tool's performance.
We thank the reviewer for these comments, which provide an opportunity to improve clarity in the manuscript. However, we respectfully disagree with the reviewer’s interpretation that MHConstructor’s “core contribution lies in using multiple MHC reference sequences to guide the assembly process”. MHConstructor does not use the six major MHC haplotypes as reference sequence from which to align the short read data for variant calling, as Reviewer #2 seems to be interpreting. We would like to clarify our use of ‘de novo’ and ‘mapping’ here. We use it in the same context as Lischer and Shimizu, 2017, the original paper whose source code and assembly algorithm are implemented in MHConstructor. Their work was designed specifically for the challenge of assembling the genome of a species that does not yet have a reference genome. They reason that the reference genome of a closely related species, while containing too much variation to meaningfully align reads to, still shares enough sequence homology with its sister species to help inform, or guide, the de novo assembly process of the novel genome. We chose the algorithm for a similar reason, since it would be sensitive to novel variation, while using the few quality MHC reference sequences that are available to inform and simplify the de novo assembly process.
‘de novo’ sequence assembly is a commonly used method of reference-free sequence assembly (Miller et al. 2010; Nagarajan and Pop 2013; Koren et al. 2018; Dida and Yi 2021; Zhang et al. 2021). It functions by breaking sequencing reads into smaller sequence fragments of length k (kmers) and then connecting kmers into graph structures based on sequence overlap (Miller et al. 2010; Nagarajan and Pop 2013).

These structures are known as DeBrujin graphs and represent the most likely path through overlapping sequence fragments (Miller et al. 2010; Nagarajan and Pop 2013).

These graphs are then condensed into longer assembled sequence fragments known as contigs. The primary benefit of this approach is that it does not rely on alignment to a reference sequence and therefore is much more likely to capture previously undescribed variation (Miller et al. 2010; Nagarajan and Pop 2013) Further, de novo assembly has already been successfully used to generate MHC sequences, examples of which have been described in the Introduction. However, none of these papers have published a reproducible pipeline or software package for the assembly process. This is one of the key motivations for creating MHConstructor. By developing a high throughput and reproducible tool for de novo MHC sequence assembly, we hope that others will be able to generate their own de novo MHC sequences as well.

To clarify the use of ‘mapping’, we only map (align) reads to the major MHC haplotypes as a means to bin (or separate) reads by haplotype. Haplotype-binning has already been successfully used in several MHC assembly contexts (Koren et al. 2018; Wang et al. 2023). We chose the set of guide MHC haplotypes that capture the primary categories of structural variation observed in the HLA class II region. As seen in Jensen et al., 2017 and Houwaart et al., 2023, this region, as well as C4 (which we account for by including C4Investigator), are the primary sources of structural variation in the MHC that have been observed thus far. Because of this, we infer that the use of these major, known categories of SVs are represented by the six major haplotypes characterized by Houwaart et al., 2023 and thus, are sufficient to assist in structural assembly of the HLA class II region. The structural variation in this region would likely thwart the de novo assembly process if assembled collectively. Therefore, we circumvent this issue by binning reads from haplotypes associated with differing structural variation prior to de novo assembly. Reads which were alignable to each haplotype are then extracted from those alignments and de novo assembled separately for each haplotype, as described in the previous paragraph. As mentioned before, this method is already published in Lischer and Shimizu, 2017.

The guide MHC haplotype sequences are then used at a second point in the MHConstructor assembly process, which is known as scaffolding. During this stage, the de novo assembled contigs are ordered and oriented with respect to a reference sequence, in this case, the guide MHC haplotypes. This is done to orient the de novo assembled contigs, not to call variation. Scaffolding is a well-established practice and the software we use to do this, RagTag, is commonly used.

Reads which did not align to a guide haplotype can be assumed to represent novel variation of a large structural nature (>150bp) and are captured through a separate de novo assembly process, only using reads which do not map to the guide haplotype. However, because of the near impossibility of meaningfully including these contigs into the final, scaffolded sequence assembly in a high throughput way, we have left these sequences out and encourage users to investigate these sequences in a post-hoc analysis that will likely require manual curation and evaluation.

We have revised the introduction and added additional clarification of these definitions to the text to make our use of haplotype binning, de novo assembly and scaffolding more easily understood. We have also added further explanation of why such an approach was chosen and the benefits it offers for accessing novel MHC variation. To demonstrate the viability of this approach for identifying novel variation not observed in the guide haplotype, we have added two additional Supplemental Tables, S9 and S10, with corresponding manuscript text added to Results 3.3 and lines 666- 677 of the Methods.

In regards to the recommendation of “comparing MHConstructor with other reference- guided methods, such as Lischer and Shimizu (2017)”, as described in the manuscript, the core, MHConstructor assembly algorithm is that of Lischer and Shimizu, 2017. We used their published bash script pipeline for our reference-guided de novo assembly and adapted it superficially to accept our specific input data and fit into the MHConstructor pipeline. Thus, it is not meaningful to compare to their method, as it is the method we used. Beyond their method, there are no other tools for reference- guided de novo assembly against which to compare our results

While we appreciate the concern for rigorous performance evaluation, we respectfully disagree with Reviewer #2’s second comment “Second, the evaluation method in sections 3.1 and 3.2, which relies on datasets associated with the reference sequences, may overestimate the tool's performance.” To clarify, we used the raw short reads from the cell lines used in both Houwaart et al., 2023 and Huijse et al., 2023 to attempt to re-create their high quality MHC reference sequences. This is a valid and meaningful way of assessing MHConstructor performance, since these high- quality reference MHC sequences were sequenced with both long and short read sequencing, and extensively manually curated for accuracy. Therefore, we used these finished sequences to represent “ground truth”. Thus, the extent to which de novo assembly with MHConstructor can reproduce these high-quality sequences from the same source data, allows for meaningful assessment of both MHConstructor’s accuracy and its errors, as well as the types of sequences that are driving its error. We have revised the relevant text at lines 650-653 to make this clearer.
As further means of validating the performance of MHConstructor, we compared the quality of MHConstructor MHC assemblies with previously published de novo MHC assemblies, from Norman et al., 2017. These data can be found in Supplemental figure S9. By comparing the distribution of contig number, N50 and assembly length, which are commonly accepted metrics for evaluating assembly quality, against a set of 95 previously published MHC assemblies (Norman et al., 2017), we find that MHConstructor performs comparably to these assemblies, and even better in terms of lower contig numbers. However, because the authors of this paper did not publish a reproducible pipelined tool, we cannot compare these methods directly on the same starting dataset.
To provide a means of validation of MHConstructor’s sensitivity to identifying novel variation, we have now added a simulation analysis, the results of which can be found in Supplemental Tabls S9 and S10, with corresponding manuscript text added to Results 3.3 and lines 666-677 of the Methods.

Other issues:
(1) Line 603-604, There are some spelling errors.
Thank you for identifying these errors. This sentence was an erroneous artifact leftover from poor edit merging during initial writing and has been removed.

(2) Line 117 "Recent work" and Line 191 "base on our previous finding" need references.
We appreciate this opportunity to clarify the manuscript. Here, “Recent work” was meant to refer back to all the of published work described and cited in earlier paragraphs of the manuscript as motivation for this study. That phrase has been removed to avoid confusion. At Lines 202-203, we have now revised it to read: “based on our findings regarding the impact of sequencing coverage depth on assembly quality, described in Section 2.1”

(3) Line 552, The item "superblock" lacks a description.
Thank you for catching this issue. This sentence has been revised to read: “Following alignment to the guide BMH, regions of mapped, overlapping reads are grouped into blocks. These blocks are then merged into “superblocks”, which represent the boundaries of adjacent, overlapping blocks, as defined in Lischer and Shimizu, 2017.”

(4) Supplemental Table S5 contains duplicate content.
Thank you for bringing our attention to this duplication. The repeated table has now been removed.

**2^nd^ round**

**Reviewer 1**

Dear authors,
I am happy to approve your manuscript. I believe that my major comments were well discussed and I am satisfied with the result. It’s a nice paper.

I allow myself to disagree on your level of description of the lab methods, which I think still lack some detail for readers to be able to replicate your work (which in my opinion is the standard that methods descriptions should meet). I also still think you oversell your pipeline when it comes to the applicability to the MHC in non-human species. There will in practice be extremely few species that have reference MHC sequences that can be used as guides – let alone a selection of reference MHC sequences that represents the haplotype variation in a wild population (which is most often completely unknown).

However, these are only minor issues and I think the community needs MHConstructor and MHConstructor needs to be used and improved with the experience of many users. I hope it will have a positive impact on MHC research.

Kind regards,
Jacob Roved
Department of Biology
Lund University

**Reviewer 2**

Thank you to the authors for the revision and adding new results, which have clarified the manuscript and addressed some of my concerns. However, I still believe that some comparisons are needed to demonstrate the importance of the contributions of MHConstructor. Firstly, the authors should compare MHConstructor with de novo short-read assembly tools and scaffolding tools designed for whole-genome assembly. This comparison is essential to demonstrate the need for developing MHC-specific assemblers. Supplemental Figure S9 displays assemblies derived from different datasets, which makes it hard to evaluate the advantages of MHConstructor. Secondly, one of MHConstructor's key contributions is its ability to select the best matching haplotype as a guide. Therefore, the evaluation is needed to determine whether this approach enhances assembly performance. The authors could compare MHConstructor with either the method of Lischer and Shimizu (2017) or with MHConstructor itself, using a fixed MHC haplotype, such as GRCh38, as the guide. I believe these comparisons will help readers better understand the advantages of MHConstructor.

**Further advice from Reviewer 1**

MHConstructor is based on the method of Lischer and Shimizu 2017, which the authors have adapted to work on the MHC region of the genome (in humans known as HLA). The MHC region poses some special challenges when it comes to assembly, especially caused by structural variation between haplotypes. They have come up with the idea of first mapping short read data to known HLA haplotypes at first field resolution (i.e., amino acid sequences) in order to bin the short reads by their haplotype associations. This step simplifies the data and makes it possible to de novo assemble the short reads into contigs, one haplotype at the time. The contigs are subsequently mapped to the guide haplotypes to generate larger scaffolds.

Conceptually, the HLA guide haplotypes incorporated in MHConstructor correspond to the reference genome employed in Lischer and Shimizu’s method. As I understand it, the authors have adopted the scripts from Lischer and Shimizu. Hence, a comparison between Lischer and Shimizu and MHConstructor is not meaningful. If you adapt Lischer and Shimizu’s method to work on MHC, you get MHConstructor. In my opinion, the authors already clearly state that they adopt Lischer and Shimizu’s method and apply it to the special case of the MHC. I think Reviewer 2 misunderstood this relationship?

As for comparisons to existing methods, there are no such methods that are specifically tailored to the MHC. The authors compare their data to previously published MHC sequences that have been produced with much more laborious methods (generated by sequencing with both long and short reads and extensive manual curation), and that’s the best they can do – such methods are currently the state of the art. In my opinion, further validation of MHConstructor has to come from actual usage of the method on real data sets.

As for comparing MHConstructor data to a fixed haplotype, I don’t think that’s possible. MHConstructor assembles de novo sequenced data, and while you can generate a known haplotype in silico, you cannot make it into a mega-base sized DNA molecule that you sequence de novo in the lab. Hence, the best representation of “ground truth” for validation is what the authors already report in Results section 3 by comparing to (a) previously published data and (b) in silico simulated short read data based on previously published sequences (L665-676 in the revised manuscript).

As for Fig. 2, that is not a comparison of MHConstructor data to reference data with the purpose of validation. Fig. 2 is an illustration of how comparing an MHConstructor assembly to a mismatching DR-haplotype generates misalignments due to structural variation. In panel A, the MHConstructor assembled haplotype A is compared to the matching DR2 haplotype, while in panel B it is compared to the mismatching DR4 type (L271-279 in the revised manuscript). Note that the validation of MHConstructor is reported in Results section 3, and specifically in tables 2 and 3 (comparison to previously published sequences) and in suppl. tables S9 and S10 (comparison to simulated short read data). The tables are sufficient in my opinion, I don’t see any need for figures illustrating those numbers.

To answer your concluding question, I don’t think further comparisons are required to validate or demonstrate the advance of MHConstructor. To quote Lischer and Shimizu 2017: “…the evaluation of genome assemblies is often not straight forward, as the truth is not known. Thus one should always use a combination of evaluation metrics, which not only try to assess the continuity but also the accuracy of an assembly.” While reviewer 2 might hold a different opinion, I believe the authors of MHConstructor fulfill this standard and provide sufficient validation of their method.

MHConstructor seems to me a good and useful method and it should get out there and be tested in real usage. Its most important advance compared to Lishcer and Shimizu is that MHConstructor is a comprehensive, containerized method that incorporates the HLA guide haplotypes that are used to bin the short reads prior to assembly.

If there are issues that can be improved, the authors can revise and update the tool in the future – but they will probably need the feedback from other users.

**Authors’ response**

We have removed the statement in the discussion referencing possible

applications to non-human species. We have also added additional details to the

methods describing the wet lab sequencing component to increase reproducibility and

provide clarity.
